# Mechanistic Insights Into the Cross-Feeding of *Ruminococcus gnavus* and *Ruminococcus bromii* on Host and Dietary Carbohydrates

**DOI:** 10.3389/fmicb.2018.02558

**Published:** 2018-11-05

**Authors:** Emmanuelle H. Crost, Gwenaelle Le Gall, Jenny A. Laverde-Gomez, Indrani Mukhopadhya, Harry J. Flint, Nathalie Juge

**Affiliations:** ^1^Quadram Institute Bioscience, Gut Microbes and Health Institute Strategic Programme, Norwich Research Park, Norwich, United Kingdom; ^2^Gut Health Group, The Rowett Institute, University of Aberdeen, Aberdeen, United Kingdom

**Keywords:** cross-feeding, gut bacteria, *Ruminococcus*, mucin, resistant starch

## Abstract

Dietary and host glycans shape the composition of the human gut microbiota with keystone carbohydrate-degrading species playing a critical role in maintaining the structure and function of gut microbial communities. Here, we focused on two major human gut symbionts, the mucin-degrader *Ruminococcus gnavus* ATCC 29149, and *R. bromii* L2-63, a keystone species for the degradation of resistant starch (RS) in human colon. Using anaerobic individual and co-cultures of *R. bromii* and *R. gnavus* grown on mucin or starch as sole carbon source, we showed that starch degradation by *R. bromii* supported the growth of *R. gnavus* whereas *R. bromii* did not benefit from mucin degradation by *R. gnavus*. Further we analyzed the growth (quantitative PCR), metabolite production (^1^H NMR analysis), and bacterial transcriptional response (RNA-Seq) of *R. bromii* cultured with RS or soluble starch (SS) in the presence or absence of *R. gnavus*. In co-culture fermentations on starch, ^1^H NMR analysis showed that *R. gnavus* benefits from transient glucose and malto-oligosaccharides released by *R. bromii* upon starch degradation, producing acetate, formate, and lactate as main fermentation end-products. Differential expression analysis (DESeq 2) on starch (SS and RS) showed that the presence of *R. bromii* induced changes in *R. gnavus* transcriptional response of genes encoding several maltose transporters and enzymes involved in its metabolism such as maltose phosphorylase, in line with the ability of *R. gnavus* to utilize *R. bromii* starch degradation products. In the RS co-culture, *R. bromii* showed a significant increase in the induction of tryptophan (Trp) biosynthesis genes and a decrease of vitamin B12 (VitB12)-dependent methionine biosynthesis as compared to the mono-culture, suggesting that Trp and VitB12 availability become limited in the presence of *R. gnavus*. Together this study showed a direct competition between *R. bromii* and *R. gnavus* on RS, suggesting that *in vivo*, the *R. gnavus* population inhabiting the mucus niche may be modulated by the supply of non-digestible carbohydrates reaching the colon such as RS.

## Introduction

The human gut is heavily populated by a diverse microbial community (gut microbiota) which plays a crucial role in maintaining human health through, e.g., polysaccharide digestion, metabolite and vitamin production, maturation of the immune system and protection against pathogens (for a review, see Thursby and Juge, 2017). The adult gut microbiota is dominated by members of Firmicutes and Bacteroidetes phyla although organisms from the Actinobacteria, Verrucomicrobia and Proteobacteria phyla also contribute to the structure and function of this microbial community. The microbiota composition varies longitudinally along the gastrointestinal tract but also transversally from the lumen to the mucosa (Donaldson et al., 2016). The colon is the most densely colonized part of the gut, reaching density of 10^11^–10^12^ cells per gram. The lumen of the gut is generally considered to host a microbial community which is distinct from that of the mucus layer although partial mixing and dispersal by host factors tend to homogenize the community (Mark Welch et al., 2017; Tropini et al., 2017). Several factors influence the biogeography of symbiotic bacteria within the gut, including the gradient and availability of glycans within discrete physical niches (Koropatkin et al., 2012).

In the colon, bacteria have access to non-digestible polysaccharides from the diet but also to complex oligosaccharides from the host mucins (Koropatkin et al., 2012; Tailford et al., 2015a). It is commonly accepted that diet is the main contributing factor influencing the structure of the gut microbial community in the colon (for a review, see Flint et al., 2017). Dietary alteration in the gut microbiota profile can be temporal (e.g., David et al., 2014) or long-term (e.g., Sonnenburg et al., 2016). One of the largest single source of energy for microbial growth in the human colon is dietary starch that escapes digestion in the upper gut and reaches the colon undigested. The fermentation of these substrates provides nutrients for the gut bacteria and short-chain fatty acids (SCFAs). SCFAs are beneficial for colon health; they are a source of energy for the colonocytes and contribute to the maintenance of gut barrier function, the protection against colorectal cancer development and the control of intestinal inflammation (Flint et al., 2017).

Ruminococcaceae are an important family of Firmicutes bacteria within the colonic microbial communities which have evolved specialized systems to utilize complex carbohydrates. This is in contrast to *Bacteroides* which have been shown to display diverse and versatile glycan metabolizing capabilities (for a recent review, see Ndeh and Gilbert, 2018). Members of the genus *Ruminococcus* have been reclassified into three genera and families based on 16S rRNA sequencing, *Blautia* (Lachnospiraceae), *Ruminococcus* (Ruminococcaceae) and *Clostridium* (Clostridiaceae) (Liu et al., 2008). *R. bromii* is one of the most abundant bacteria constituting the human colonic microbiota and a primary degrader of RS, an important non-digestible dietary polysaccharide (Ze et al., 2012, 2013). *Ruminoccocus gnavus* was first assigned as a novel species in 1976 (Moore et al., 1976) and recently reclassified into genus *Blautia* which belongs to *Clostridium* cluster XIVa, a member of the Lachnospiraceae family but still maintaining its original name (Lawson and Finegold, 2015). *R. bromii* and *R. gnavus* are prevalent species of the human gut; They are among the 57 species detected in more than 90% of human fecal samples by metagenomic sequencing (Qin et al., 2010). The median abundances of *R. bromii* L2-63 and *R. gnavus* are around 3 and 0.1%, respectively (Qin et al., 2010). In our previous work we showed that *R. gnavus* ability to grow on host mucin glycoproteins was strain dependent (Crost et al., 2013, 2016), underscoring the importance of analyzing glycan utilization by members of the human gut microbiota at the strain level. The mucin-degrading strain *R. gnavus* ATCC 29149 utilizes mucin glycan epitopes from the intestinal mucus layer as energy source (Crost et al., 2013, 2016).

It has been proposed that the primary role played by *R. bromii* is to release energy from RS to other members of the microbial community (Ze et al., 2012). Trophic interactions between members of the microbiota encompass both cooperation and competition. For example, mucin cross-feeding has been reported between gut microbiota species such as infant bifidobacteria and *Eubacterium hallii* (Bunesova et al., 2018) or *Akkermansia muciniphila* and non-mucus-degrading bacteria *Anaerostipes caccae*, *Eubacterium hallii*, or *Faecalibacterium prausnitzii* (Belzer et al., 2017; Chia et al., 2018). Examples of cross-feeding have also been reported within the *Bifidobacterium* genus (Milani et al., 2015; Turroni et al., 2017), and in the presence of primary degraders of RS (with *R. bromii*) or xylan (with *B. ovatus*) (Turroni et al., 2010, 2012; Rogowski et al., 2015; Centanni et al., 2017).

Here, we investigated the molecular mechanisms underpinning the trophic interactions between the human gut symbionts *R. bromii* and *R. gnavus* on host mucin and dietary starch using a combination of bioinformatics, quantitative PCR (qPCR), NMR-based metabolite profiling and RNA-Seq based transcriptomics of mono- and co-cultures.

## Materials and Methods

### Materials

D-glucose (Glc), type III pig gastric mucin (PGM), maltose, maltotriose and soluble potato starch (SS) were purchased from Sigma-Aldrich (St Louis, MO, United States). Purified pig gastric mucin (pPGM) was prepared as previously described (Gunning et al., 2013). Maltotetraose was obtained from Carbosynth (Berks, United Kingdom). A retrograded type-III RS derived from high amylose maize was kindly provided by Ingredion (Manchester, United Kingdom).

### Bacterial Strains and Growth Conditions

*Ruminococcus gnavus* ATCC 29149 was routinely grown in an anaerobic cabinet (Don Whitley, Shipley, United Kingdom) in Brain Heart Infusion broth supplemented with yeast extract and hemin (BHI-YH) as previously described (Crost et al., 2013). *R. bromii* L2-63 was also grown in an anaerobic cabinet, in anaerobic basal Yeast extract-Casitone-Fatty Acids (YCFA) medium (Duncan et al., 2002) supplemented with 0.5% SS. Growth of both bacteria on single carbon sources utilized YCFA medium supplemented with 0.5% (wt/vol) of Glc or starch (SS or RS), malto-oligosaccharides at a concentration of 27.7 mM Glc units, or 1% (wt/vol) pPGM. The growth assays were performed in 96-well plates with 200 μL of medium/well for screening or in 14 mL-tubes with 10 mL medium/tube for sampling. Growth was determined spectrophotometrically by monitoring changes in optical density (OD) at 595 nm or 600 nm compared to the same medium without bacterium (ΔOD_600__nm_) hourly for the first 10 h and then at distinct times up to 75 h. Sampling for DNA extraction, RNA extraction or ^1^H NMR was carried out over growth.

### DNA Extraction and qPCR

For the isolation of *R. gnavus* ATCC 29149 and/or *R. bromii* L2-63 chromosomal DNA, cells from a 2 mL-aliquot of culture were harvested by centrifugation (10,000 g, 5 min, 4°C), at different times of growth. The cell pellet was kept frozen at −20°C until DNA extraction. The DNA extraction was carried out using Gene JET Genomic DNA Purification kit (ThermoFisher Scientific) following the supplier’s procedure for Gram-positive bacteria, except for the elution step which was performed with 100 μL of EB buffer instead of 200 μL. DNA quality and quantity were assessed using the NanoDrop^TM^ 2000 spectrophotometer (ThermoFisher Scientific) and the Qubit dsDNA HS assay on Qubit^®^ 2.0 fluorometer (ThermoFisher Scientific). Dilutions at 10 ng/μL were prepared in water then the DNA was diluted further in 5 μg/ml Salmon Sperm DNA to obtain a 1 ng/μL dilution used as template for qPCR (see below).

The 16S rRNA genes of *R. gnavus* ATCC 2949 and *R. bromii* L2-63 were amplified with universal primers 27F (5″- AGAGTTTGATCMTGGCTCAG- 3″) and RP2 (5″-ACGGCTACCTTGTTACGACTT-3″). The PCR products were purified, quantified and diluted in water to 16.4 ng/μL which equals to 10^10^ copies/μL. A series of 10-fold or 20-fold dilutions was then performed from 10^10^ copies/μL to 10^2^ copies/2 μL using 5 μg/mL Salmon Sperm DNA. Calibration curves were prepared in triplicates for each pair of primers using 10^7^ copies/2 μL to 10^2^ copies/2 μL dilutions of 16S PCR products. The standard curves showed a linear relationship of log input 16S copy number vs. the threshold cycle (C_T_), with acceptable values for the slopes and the regression coefficients (R^2^). The dissociation curves were also performed to verify the specificity of the amplicons.

Quantitative PCR was carried out in an Applied Biosystems 7500 Real-Time PCR system (Life Technologies Ltd). Three pairs of primers targeting 16S rRNA gene were used in this study (Supplementary Table S1). Each qPCR reaction (10 μL) was then carried out in triplicates with 2 μL of DNA matrix at 1ng/μL and 0.2 mM of each primer, using the QuantiFast SYBR Green PCR kit (Qiagen) according to supplier’s advice (except for the combined annealing/extension step which was extended to 35 s instead of 30 s).

### RNA Extraction, Ribodepletion and RNA-Seq

Total RNA was extracted from 5 mL of mid- to late exponential phase cultures of *R. gnavus* ATCC 29149 and/or *R. bromii* L2-63 in YCFA supplemented with a single carbon source (Glc, SS or RS). Four biological replicates were performed for each carbon source. The RNA was stabilized prior to extraction by adding 1/5 vol of phenol (pH 4.3): ethanol (1:9) mixture to 1 vol of culture then incubating 30 min on ice and finally pelleting the cells for 5 min at 10,000 g at 4°C. Cell pellets were stored at −80°C before extraction. The extraction was performed by a method using phenol and chloroform and adapted from Sambrook et al. (1989). Genomic DNA contamination was removed by DNAse treatment using the TURBO DNA-free kit (Life Technologies Ltd., Paisley, United Kingdom) according to the supplier’s recommendations. The purity, quantity and integrity of the DNase-treated RNA were assessed with NanoDrop 2000 Spectrophotometer and with Agilent RNA 600 Nano kit on Agilent 2100 Bioanalyzer (Agilent Technologies, Stockport, United Kingdom). Ribodepletion was then carried out using Ribo-Zero rRNA Removal kit for bacteria according to supplier’s advice (Illumina, Cambridge, United Kingdom); efficiency assessment of the ribodepletion was performed by quantifying RNA before and after rRNA removal using the Qubit RNA HS assay on Qubit 2.0 fluorometer.

The rRNA removal was confirmed with a Nano chip run on a Bioanalyzer 2100 (Agilent). Three out of the four replicates were selected for sequencing for each condition. The resulting ribosomal depleted RNA was then fragmented for 8 min at 94°C using the Elute, Fragment, Prime buffer from Illumina TruSeq RNA kit. These conditions produced final libraries of around 370 bp. The samples were then processed following the standard TruSeq RNA protocol. The 15 Illumina libraries were normalized and equimolar pooled to 11 nM using elution buffer (Qiagen) and run over two lanes of the Illumina HiSeq2500 with a 100 bp paired end read metric.

The library pool was then diluted to 2 nM with NaOH and 5 μL transferred into 995 μL HT1 (Illumina) to give a final concentration of 10 pM. A portion (120 μL) of the diluted library pool was then transferred into a 200 μL-strip tube, spiked with 1% PhiX Control v3 and placed on ice before loading onto the Illumina cBot. The flow cell was clustered using a HiSeq PE Cluster Kit v3 (Illumina PE-401-3001) utilizing the Illumina PE_HiSeq_Cluster_Kit_V3_cBot_recipe_V8.0 method on the Illumina cBot. Following the clustering procedure, the flow-cell was loaded onto the Illumina HiSeq2500 instrument following the manufacturer’s instructions with a 101 cycle paired reads and a 7-cycle index read. The sequencing chemistry used was HiSeq SBS Kit v3 (Illumina FC-401-3001) with HiSeq Control Software 2.2.68 and RTA 1.18.66.3. Reads in bcl format were demultiplexed based on the 6 bp Illumina index by CASAVA 1.8, allowing for a one base-pair mismatch per library, and converted to FASTQ format by bcl2fastq. The RNA-Seq reads were aligned against the combined reference of *Ruminococcus_bromii*_l2_63.ASM20987v1.31.dna.genome.fa and *Ruminococcus_gnavus*_atcc_29149.ASM16947v1.31.dna.genome.fa using tophat v2.1.0 with the –max-multihits 1 option. Read counts were obtained using htseq-count v0.6.1^1^. The differential expression analysis was carried out using the DESeq2 (v1.14.0) package (Love et al., 2014). The transcript counts were used as input for DESeq2 and filtered to remove any transcripts with a total count of 0 or 1 over all the samples. Raw counts were normalized to the effective library size separately for *R. bromii* and *R. gnavus* before carrying out the differential expression analysis using the DESeq function. An padj cut-off of 0.05 was used to determine differential expressed transcripts.

### ^1^H Nuclear Magnetic Resonance Analysis (^1^H NMR)

^1^H NMR analysis was used to identify the presence, absence, and concentration of several metabolites in the bacterial growth medium of mono- and co-cultures. The spent media were thawed at room temperature and prepared for ^1^H NMR spectroscopy by mixing 400 μL of spent medium with 200 μL of phosphate buffer (0.26 g NaH_2_PO_4_ and 1.41 g K_2_HPO_4_) made up in 100% D_2_O (100 mL), containing 0.1% NaN_3_ (100 mg), and 1 mM sodium 3-(Trimethylsilyl)-propionate-*d*4, (TSP; 17 mg) as a chemical shift reference. The samples were mixed, and 500 μL was transferred into a 5-mm NMR tube for spectral acquisition. The ^1^H NMR spectra were recorded at 600 MHz on a Bruker Avance spectrometer (Bruker BioSpin GmbH, Rheinstetten, Germany) running Topspin 2.0 software and fitted with a cryoprobe and a 60-slot autosampler. Each ^1^H NMR spectrum was acquired with 512 scans, a spectral width of 12300 Hz and an acquisition time of 2.7 s and delay time of 3 s. The “noesygppr1d” presaturation sequence was used to suppress the residual water signal with a low-power selective irradiation at the water frequency during the recycle delay. Spectra were transformed with a 0.3-Hz line broadening, manually phased, baseline corrected, and referenced by setting the TSP methyl signal to 0 ppm. Metabolites were identified using information found in the Human Metabolome Database^2^ and by use of the 2D-NMR methods, COZY, HSQC, and HMBC. The metabolites were quantified using the software Chenomx NMR suite 7.6^TM^.

### Bioinformatics Analysis

Genome mining was performed manually by BLAST using the “align two or more sequences” tool (Boratyn et al., 2013). For each target protein, the query sequence used was the reference protein sequence from NCBI; when no reference protein sequence was available, a sequence from a member of the Clostridiales order was used (Supplementary Table S2). The subject sequences were the sequences corresponding to all putative proteins from *R. bromii* L2-63 or *R. gnavus* ATCC 29149 genomes. The search for NanA, E and K in *R. bromii* L2-63 was performed according to Almagro-Moreno and Boyd (Almagro-Moreno and Boyd, 2009).

## Results and Discussion

### *R. gnavus* Utilizes Starch Degradation Products Released by *R. bromii*

**FIGURE 1:**
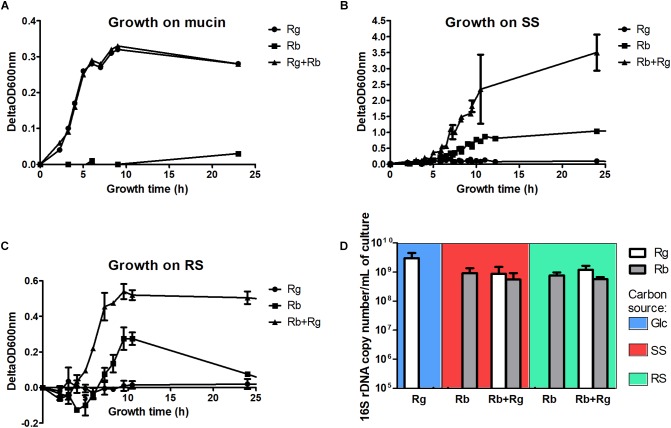
Growth curves of the mono- and co-cultures with mucin **(A)**, soluble starch (SS) **(B)** or resistant starch (RS) **(C)** as sole carbon source and cell concentrations in the different growth conditions **(D)**. The concentrations were determined by qPCR and expressed as 16S rDNA copy number/mL of culture. The values are averages of 3 replicates for *R. gnavus* ATCC 29149 grown with Glc or 4 replicates for the other conditions. The error bars correspond to the standard deviations. Cells samples were collected at a time of growth of 7 h for *R. gnavus* ATCC 29149 grown on Glc, 10 h for *R. bromii* L2-63 grown on SS and 8 h for the other conditions.

The trophic interactions between *R. bromii* L2-63 and *R. gnavus* ATCC 29149 on host and dietary carbon sources were determined under anaerobic conditions using YCFA as a suitable minimum medium for both strains (Duncan et al., 2002). The growth of *R. bromii* L2-63 and *R. gnavus* ATCC 29149 in mono- or co-cultures was first monitored spectrophotometrically using mucin as sole carbon source. While *R. gnavus* could utilize this substrate, as previously reported (Crost et al., 2013)*, R. bromii* was unable to grow on mucin as sole carbon source and no growth benefit was observed on this substrate in the presence of *R. gnavus* (Figure 1A). Mucin degradation by bacteria relies on the expression of glycoside hydrolases (GHs)^2^ (Lombard et al., 2014) such as sialidases (GH33), α-fucosidases (GH29, GH95), exo- and endo-β-N-acetylglucosaminidases (GH84 and GH85), β-galactosidases (GH2, GH20, GH42), α-N-acetylglucosaminidases (GH89), endo-β1,4-galactosidases (GH98) or α-N-acetylgalactosaminidases (GH101 and GH129) (Tailford et al., 2015a). We previously showed that the ability of *R. gnavus* to grow on mucin was dependent on the expression of a GH33 intramolecular *trans*-sialidase (Crost et al., 2013, 2016; Tailford et al., 2015b) and that fucose was released from mucin by the action of GH29 and GH95 fucosidases (Crost et al., 2013). In contrast, the *R. bromii* L2-63 genome encodes a small number of GHs (Mukhopadhya et al., 2018) compared to *R. gnavus* ATCC 29149 (Crost et al., 2013) (21 in *R. bromii* L2-63 vs. 60 in *R. gnavus* ATCC 29149) and no genes encoding mucin-degrading enzymes were found, in line with the inability of this strain to grow on mucin. In addition, its lack of growth in co-culture with *R. gnavus*, suggests that *R. bromii* cannot utilize the monosaccharides released by *R. gnavus*, in agreement with genomic data suggesting that *R. bromii* does not harbor genes involved in fucose or sialic acid metabolism (Supplementary Figures S1, S2).

*Ruminococcus bromii* L2-63 is highly specialized in starch degradation, dedicating 15 of its 21 GH-encoding genes to putative GH13 amylases (Ze et al., 2015). Some of these GH13 amylases revealed an organization in “amylosome”, contributing to *R. bromii* exceptional ability to degrade dietary RS (Ze et al., 2015; Mukhopadhya et al., 2018). Here we showed that *R. bromii* was able to utilize both starch substrates (SS and RS) as sole carbon source, in agreement with previous reports (Ze et al., 2012, 2015) whereas no growth was detected with *R. gnavus* on these substrates despite the presence of 9 GH13-encoding genes in *R. gnavus* ATCC 29149 genome. Furthermore, while *R. bromii* growth on SS reached ΔOD_600__nm_ of ∼0.8–1, after 12 h of growth, the presence of *R. gnavus* increased the density of cells to ΔOD_600__nm_ ∼ 4 after 10 h, suggesting cross-feeding activity (Figure 1B). Due to the presence of insoluble RS particles, the OD_600__nm_ measurements of *R. bromii* grown on RS result in a two-stage curve reflecting both bacterial growth and bacterial degradation of the RS particles. When *R. gnavus* and *R. bromii* were co-cultured with RS, a different profile was observed (Figure 1C), suggesting that cross-feeding also occurs on RS, as confirmed below.

To further assess the behavior of *R. bromii* L2-63 and *R. gnavus* ATCC 29149 on starch (SS or RS), the bacteria were quantified by determining 16S rDNA copies per mL of culture by qPCR (Figure 1D). The average 16S rDNA copies of *R. bromii* when grown in mono- or co-cultures with RS after 8 h was 7.3 × 10^8^ and 5.6 × 10^8^ per mL of culture, respectively. When SS was used as the carbon source, *R. bromii* 16S rDNA copies per mL of culture was 1 × 10^9^ after 10 h in mono-culture and 5.8 × 10^8^ after 8 h in co-culture with *R. gnavus.* These analyses indicate that the presence of *R. gnavus* did not affect *R. bromii* growth on starch (RS or SS). *R. gnavus* reached high concentration level in both SS and RS co-cultures with 16S rDNA copies/mL of 6.7 × 10^8^ and 1.2 × 10^9^, respectively, while it was not able to grow in mono-culture on these substrates, confirming that *R. gnavus* benefits from *R. bromii* starch degradation by cross-feeding, as also suggested by spectrophotometric measurements. These concentrations were comparable to the growth of *R. gnavus* on 0.5% Glc as sole carbon reaching 3 × 10^9^ 16S rDNA copies/mL after 7 h of growth (Figure 1D).

**FIGURE 2:**
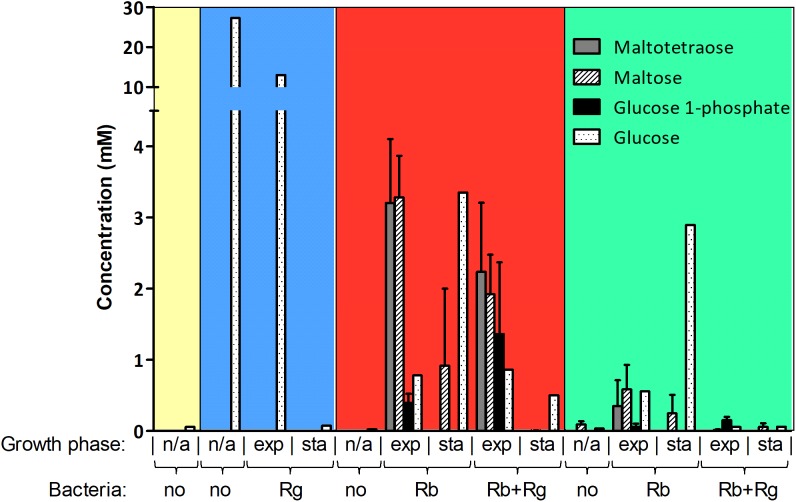
Concentration of starch degradation products in the spent media. The concentrations were determined by ^1^H NMR and the values are averages of 2 to 5 replicates. The error bars correspond to standard deviations. Results presented in the blue, red and green boxes come from growth assays with Glc, SS and RS as sole carbon source, respectively. Results from the YCFA medium alone, without carbon source, are presented inside the yellow box. Abbreviations: exp, exponential; sta, stationary; n/a, non-applicable.

The production and utilization of starch degradation products was monitored over time by ^1^H NMR (Figure 2). Maltotetraose, maltose and glucose-1-phosphate were detected in the spent medium of *R. bromii* mono- or co-cultures (with SS or RS) and their concentration decreased over time. However, while maltotetraose and glucose-1-phosphate were only detected during the exponential phase, maltose was still present at the late stage of growth in mono-cultures. Glc was also released by *R. bromii* degradation of SS or RS but tended to accumulate in mono-cultures while its concentration decreased over time in the presence of *R. gnavus*. Interestingly, the concentration of these starch degradation products was higher in *R. bromii* mono-culture on SS as compared to RS, which may be due to a slower rate of RS degradation allowing a more efficient uptake of the products.

In order to determine which starch degradation products were utilized by *R. bromii* and *R. gnavus*, mono-cultures were performed with malto-oligosaccharides and Glc as control. Both strains could utilize maltose, maltotriose and maltotetraose while Glc was only a substrate for *R. gnavus* (data not shown), suggesting that the release of Glc and malto-oligosaccharides upon *R. bromii* starch degradation contributed to *R. gnavus* cross-feeding on SS or RS. These results suggest that both syntrophy and competition could take place when *R. gnavus* and *R. bromii* are co-cultured with starch.

### Effect of Starch Co-cultures on Bacterial Metabolism

**FIGURE 3:**
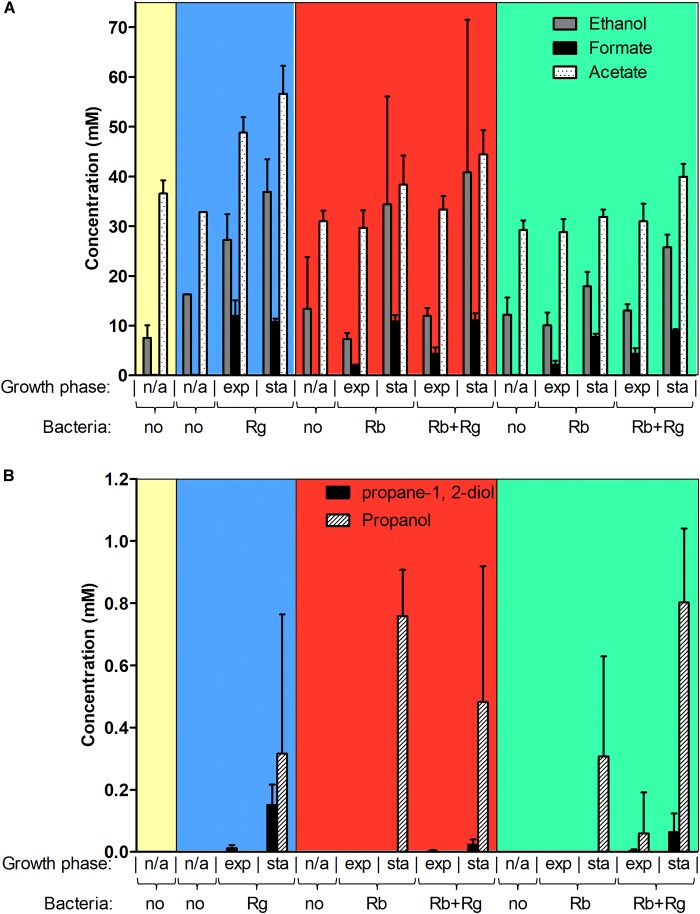
Concentration of different metabolites in the spent media. Concentrations of ethanol, formate and acetate are shown in panel **(A)** while concentrations of propane-1, 2-diol and propanol are shown in panel **(B)**. These concentrations were determined by ^1^H NMR and the values are averages of 2 to 5 replicates. The error bars correspond to standard deviations. Results presented in the blue, red and green boxes correspond to growth assays with Glc, SS and RS as sole carbon source, respectively. Results from the YCFA medium alone, without carbon source, are presented inside the yellow box. Abbreviations: exp, exponential; sta, stationary; n/a, non-applicable.

Next, we determined the metabolites produced by the bacteria in mono- and co-culture by ^1^H NMR analysis of the spent media. Acetate was the main SCFA produced by both *R. bromii* L2-63 and *R. gnavus* ATCC 29149 in mono- or co-cultures and its production was increased during bacterial growth (Figure 3A). No butyrate or propionate was detected in the growth conditions tested. Formate and ethanol were produced in increasing amounts by both *R. bromii* L2-63 and *R. gnavus* ATCC 29149 in mono- or co-cultures during bacterial growth (Figure 3A). Propanol was detected at low concentration at the late stage of growth when *R. gnavus* was grown with Glc as well as when *R. bromii* was grown with starch in mono- or co-cultures (Figure 3B). Interestingly, propanediol was only produced when *R. gnavus* was present (in mono-culture with Glc or in co-cultures with starch), suggesting that propanol is produced via different pathways in *R. bromii* L2-63 and *R. gnavus* ATCC 29149 (Figure 3B).

Three main propanol biosynthesis pathways have been identified in bacteria, the propane-1, 2-diol pathway, the acrylate pathway and the Wood-Werkman cycle (Reichardt et al., 2014). These pathways all share the last step, i.e., the conversion of propanal into propanol catalyzed by a propanol dehydrogenase (PduQ). Search for putative enzymes involved in propanol production in *R. gnavus* ATCC 29149 and *R. bromii* L2-63 genomes, identified genes encoding putative PduQ proteins, RUMGNA_01033 and L2-63_01124, respectively. No gene encoding a putative acryloyl-CoA reductase could be found in the genome of these strains, ruling out the acrylate pathway for propanol production in these bacteria. Although both bacteria encode a putative lactaldehyde reductase and a propanol dehydrogenase, PduCDE homologs could only be found in the *R. gnavus* genome indicating that propanol can be produced via the propane-1, 2-diol pathway in this bacterium. This pathway is involved in metabolism of the deoxy-sugars fucose and rhamnose (Reichardt et al., 2014). In addition to PduQ, both bacteria encode homologs of the methylmalonyl-CoA carboxytransferase and propanal dehydrogenase needed for propanol production via the Wood-Werkman cycle (Supplementary Figure S3).

### Effect of Starch Co-cultures on Bacterial Transcription

To gain further insights into the metabolic pathways underpinning trophic interactions between the two strains, transcriptional analyses of *R. bromii* L2-63 and *R. gnavus* ATCC 29149 grown in mono-cultures on starch (RS or SS) or Glc, respectively, or co-cultures on starch (RS or SS) were performed by RNA-Seq. An average of 20 million reads were generated for each sample which is sufficient sequencing depth. In the co-cultures, the reads assigned to *R. bromii* represented on average around 42% of total reads, for both SS and RS. This result correlates well with the bacterial count determined by qPCR where *R. bromii* 16S copies represented 46% and 32% of total 16S copies in SS and RS, respectively. Differential gene expression analysis (DESeq 2) was carried out to determine the influence of the carbon source or of the other bacterium on gene transcription.

**FIGURE 4:**
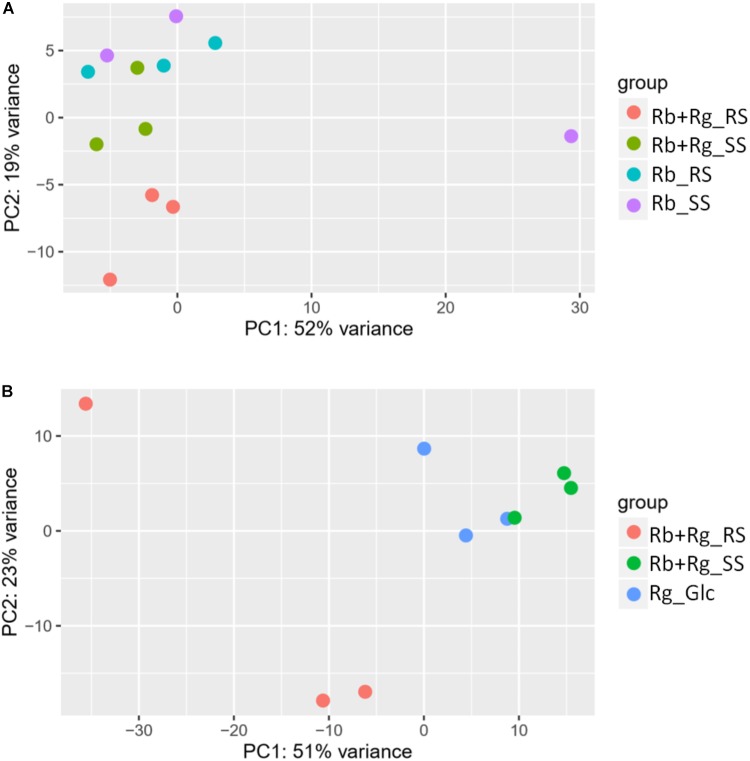
Principal component analysis (PCA) plots for transcriptomics data of *R. bromii* L2-63 genes **(A)** and *R. gnavus* ATCC 29149 genes **(B)**.

**FIGURE 5:**
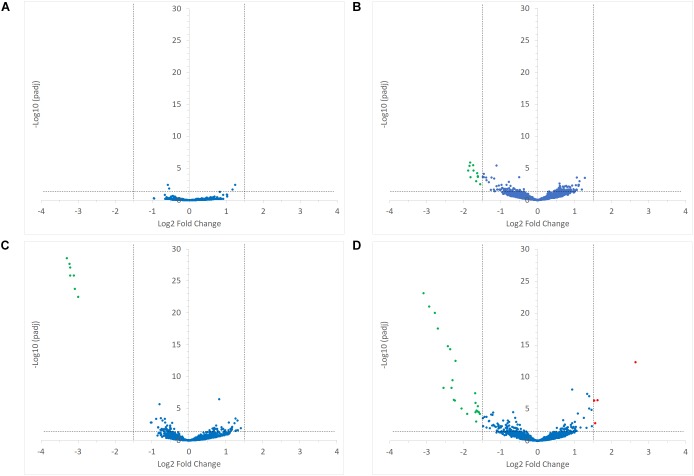
Volcano plots representing the differential expression analysis of *R. bromii* L2-63 genes. Genes are considered to be differentially expressed when Log2 Fold Change < –1.5 or > 1.5 and padj < 0.05; non-differentially expressed genes are shown as blue dots. The impact of starch on *R. bromii* L2-63 gene transcription in mono-cultures and co-cultures is shown in panel **(A,B)**, respectively. **(A)** No gene was differentially expressed between both mono-cultures. **(B)** When comparing the co-cultures, 11 genes were up-regulated with RS as compared to SS (shown as green dots). The impact of *R. gnavus* ATCC 29149 on *R. bromii* L2-63 gene transcription with SS and RS as sole carbon source is shown in panel **(C,D)**, respectively. **(C)** When SS was used as carbon source, 7 genes were up-regulated in the co-culture as compared to the mono-culture (shown as green dots). **(D)** When RS was used as carbon source, 23 genes were up-regulated in the co-culture (shown as green dots) while 4 genes were upregulated in the mono-culture (shown as red dots).

**FIGURE 6:**
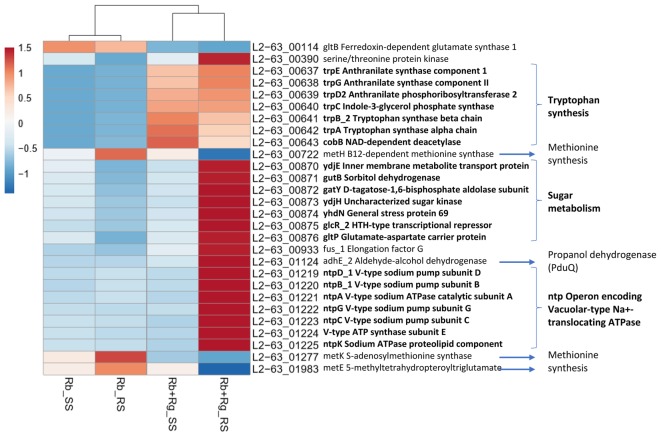
Heatmap of the transcription level (in arbitrary unit) of differentially expressed (Log2 Fold Change < –1.5 or > 1.5 and padj < 0.05) *R. bromii* L2-63 genes in different growth conditions. This heatmap was produced with ClustVis web tool (Metsalu and Vilo, 2015) using the transcript counts as input values.

Interestingly, the transcription of all *R. bromii* genes was found to be very similar in both mono-cultures irrespective of the type of starch (Figures 4A, 5A), suggesting that the catabolism of RS or SS shares the same metabolic pathway. For example, the dockerin-carrying amylases Amy4, Amy9, Amy10, and Amy12 GH13 enzymes (Ze et al., 2015) were all expressed in the conditions tested in this study. However, the type of starch had an impact on *R. bromii* gene transcription when in co-culture with *R. gnavus*, with 11 genes up-regulated with RS compared to SS, suggesting a combined effect of RS and *R. gnavus* (Figure 5B). These genes belong to 3 different clusters: 3 are part of a cluster of genes potentially involved in sugar metabolism, one is the *pduQ* gene which is involved in the conversion of propanal into propanol (see above) and 7 genes (ntpABCDEGK) are involved in the formation of a ntp sodium pump operon encoding Vacuolar-type Na + -translocating ATPase (Figure 6 and Supplementary Table S3). Interestingly, sodium and potassium ion gradients also serve as important energy reservoirs of bacterial cells and could be upregulated due to the competitive stress in the co-culture (Murata et al., 1996).

In co-cultures, *R. gnavus* had a greater effect than the type of starch on *R. bromii* gene expression. (Figures 5C,D, 6 and Supplementary Table S3). Seven *R. bromii* genes were upregulated in the presence of *R. gnavus* irrespective of the carbon source (Figure 6 and Supplementary Table S3); these genes, which include trpA, B, C, D, E and G, are all involved in the tryptophan biosynthetic pathway and are expressed when tryptophan level is low. In particular trpA, B, C, D, E and G genes were increased by around 13-fold and ninefold in the co-cultures with SS and RS, respectively, as compared to the corresponding mono-cultures. Tryptophan is metabolized by enzymes in the gut mucosa and also by enzymes produced by the gut microbiome. In *R. gnavus* ATCC 29149, RUMGNA_01526 is capable of decarboxylating tryptophan to tryptamine, an activity that is rare among bacteria, and also shared by the common gut Firmicutes member, *Clostridium sporogenes* ATCC 15579 (Williams et al., 2014). It is estimated that ∼10% of the human population harbor one of these enzymes. Interestingly, *R. gnavus* genes involved in tryptophan biosynthesis were not differentially expressed between the three conditions tested, which may be due to *R. gnavus* higher capacity to acquire and metabolize tryptophan from the medium. NMR data confirmed that the tryptophan level in the spent medium was lower in the co-cultures as compared to the mono-cultures (data not shown). Together these data suggest that tryptophan may become a limiting factor for *R. bromii* growth on this substrate in the presence of *R. gnavus*. In addition, 16 genes were found to be specifically upregulated in the RS co-culture as compared to the RS mono-culture, including 4 genes belonging to a cluster of genes involved in sugar metabolism (Figure 6 and Supplementary Table S3). It is worth noting that 10 of the 16 genes were also up-regulated in the RS co-culture as compared to SS co-culture. These results further indicate that the observed transcriptional changes in *R. bromii* L2-63 were due to a combined effect of *R. gnavus* and RS.

Interestingly, *R. bromii* showed a down-regulation of the vitamin B12-dependent methionine synthesis genes (metE, metH, metK) in the RS co-culture as compared to the RS mono-culture (Figure 5D). The downregulation in co-culture could be due to the lack of sufficient VitB12 (cobalamin) amount in the YCFA growth medium to sustain both *R. bromii* and *R. gnavus* growth as *R. bromii* does not have the ability to produce this vitamin (Ze et al., 2015).

The transcription profile of *R. bromii*, indicate the requirement for *R. bromii* to adjust its metabolic activity toward tryptophan and vitamin B12, especially when RS was used as sole carbon source, so that its growth remains unaffected in the presence of *R. gnavus* as shown above.

**FIGURE 7:**
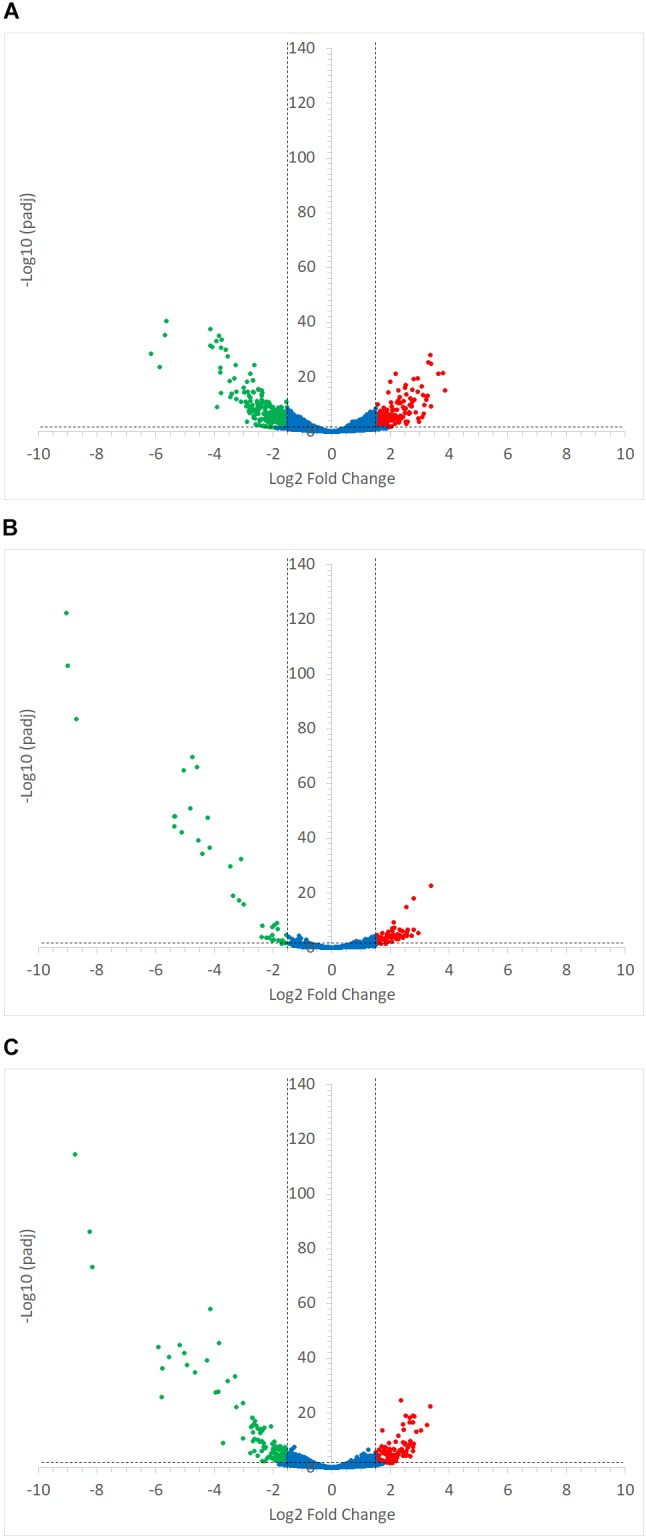
Volcano plots representing the differential expression analysis of *R. gnavus* ATCC 29149 genes. Genes were considered to be differentially expressed when Log2 Fold Change < –1.5 or > 1.5 and padj < 0.05; non-differentially expressed genes are shown as blue dots. Panel **(A)** shows the impact of starch type on *R. gnavus* ATCC 29149 gene transcription when co-cultured with *R. bromii* L2-63; 213 genes were upregulated in the co-culture with RS (shown as green dots) while 212 genes were up-regulated in the co-culture with SS (shown as red dots). The combined effect of the presence of *R. bromii* L2-63 and the carbon source (starch vs. glucose) is shown in panels **(B)** and **(C)** when SS or RS was used in the co-culture, respectively; **(B)** When SS was used as carbon source, 40 genes were up-regulated in the co-culture (shown as green dots) and 59 genes were up-regulated in the mono-culture (shown as red dots). **(C)** When RS was used as carbon source, 119 genes were up-regulated in the co-culture (shown as green dots) while 101 genes were up-regulated in the mono-culture (shown as red dots).

**FIGURE 8:**
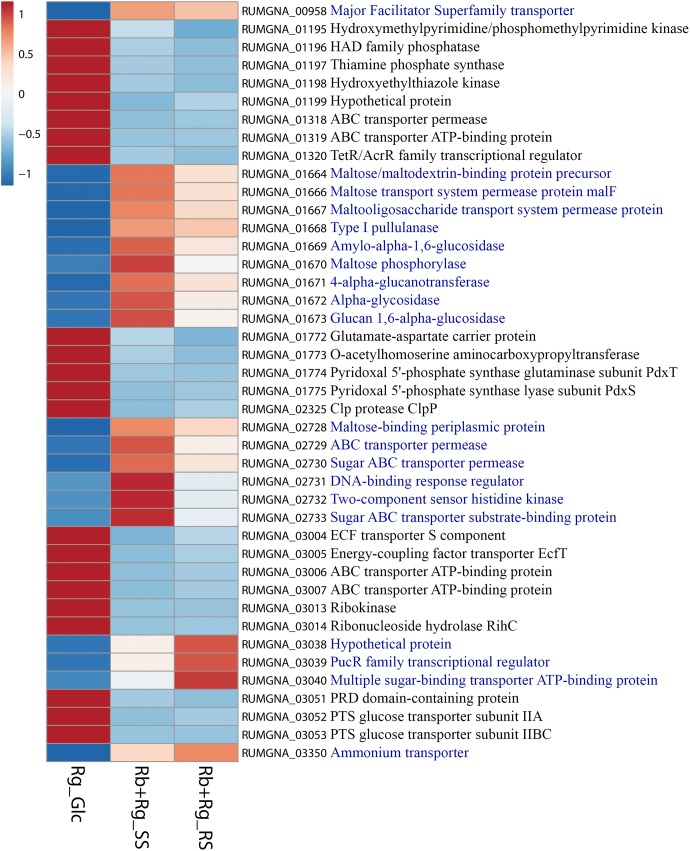
Heatmap of the transcription level (in arbitrary unit) of selected differentially expressed (Log2 Fold Change < –1.5 or > 1.5 and padj < 0.05) *R. gnavus* ATCC 29149 genes in different growth conditions. This heatmap was produced with ClustVis web tool (Metsalu and Vilo, 2015) using the transcript counts as input values. The 20 *R. gnavus* ATCC 29149 genes with an upregulated transcription in both co-cultures with *R. bromii* L2-63 on starch compared to the mono-culture on Glc are in blue. The 22 *R. gnavus* ATCC 29149 genes with an upregulated transcription in the mono-culture on Glc compared to both co-cultures with *R. bromii* L2-63 on starch are in black.

*Ruminococcus gnavus* ATCC 29149 showed a much higher number of differentially expressed genes between mono- and co-cultures as compared to *R. bromii* (Figure 7), especially with RS (Figure 4B). A total of 22 genes were down-regulated in both co-cultures compared to the mono-culture whereas 20 were upregulated including genes encoding several maltose transporters and enzymes involved in degradation of starch-related products such as RUMGNA_01664 to 01673 and RUMGNA_02728 to 02733 (Figure 8 and Supplementary Table S4). These results are in agreement with the NMR findings showing that *R. gnavus* can utilize malto-oligosaccharides as sole carbon source and from the qPCR analysis showing that *R. gnavus* can efficiently cross-feed on starch-degradation products when grown with *R. bromii*. The fact that, upon *R. bromii* starch degradation, *R. gnavus* benefits from Glc (which is not a substrate for *R. bromii*) may explain why *R. bromii* transcription is not affected on SS (a rapidly degradable starch) in co-culture as this will serve as a preferential nutrient source for *R. gnavus*. However, the ability of *R. gnavus* to utilize malto-oligosaccharides, which is a major nutrient source for *R. bromii* suggests a direct competition between the two strains, which is reflected by *R. bromii* transcriptomics data on RS. This is corroborated by the results of starch degradation products in the spent media (Figure 2) which showed Glc presence in SS co-culture medium compared to the absence of Glc in the slow degrading RS co-culture medium.

In summary, we showed that, *in vitro*, *R. gnavus* can efficiently cross-feed on starch degradation products released by *R. bromii*. Cross-feeding plays a crucial role in microbial community shaping in the gut (Hoek and Merks, 2017). This concept involves the ability of bacteria to benefit from substrate degradation products but also from fermentation products and/or cofactors. For example, *Anaerostipes caccae* L1-92 can utilize both mucin sugars and acetate produced by mucin degradation by *Akkermansia muciniphila* ATCC BAA-835 to sustain its growth and produce butyrate (Chia et al., 2018). Here, we showed that *R. bromii* L2-63 could not benefit from degradation products or metabolites released by *R. gnavus* ATCC 29149 grown on mucin, in line with the unique genomic characteristics of *R. bromii* strains sequenced to date (Mukhopadhya et al., 2018) and the mucin foraging profile of *R. gnavus* strains (Crost et al., 2013, 2016). However, we showed that RS cross-feeding initiated by *R. bromii* promoted growth of *R. gnavus* leading to the concomitant production of acetate as the main SCFA produced by these strains. Cross-feeding of gut bacteria on starch degradation products has previously been reported *in vitro* between starch degrader *R. bromii* or *Bifidobacterium longum* subsp. *suis* and bacterial species, potentially sharing the same nutrient niche in the gut such as *Anaerostipes hadrus* (Ze et al., 2013) or *B. thermacidophilum* subsp. *porcinum* (Milani et al., 2015), respectively. Resource sharing is an important ecological feature of microbial communities living in the gut (Tannock et al., 2012; Pereira and Berry, 2017; Centanni et al., 2018). The findings from our study suggest that, although *R. gnavus* strains are adapted to the mucosal environment owing to their mucin-foraging capacity (Crost et al., 2013, 2016; Tailford et al., 2015b; Owen et al., 2017), their population dynamics within the colon may also be affected by the supply of dietary carbohydrates that reaches the large intestine undigested such as RS. Due to the high prevalence of *R. bromii* in the human colon, the hydrolysis of RS will cause the release of nutrients such as glucose or metabolites that may reach bacterial species within the mucus layer, potentially promoting the growth of other species to occur, thereby further underscoring the role of *R. bromii* as a keystone species. These findings open the door to future efforts to explore cross-feeding activities between different nutrient niches *in vivo* and the use of RS or other complex polysaccharides as a strategy to address dysbiosis of mucus-associated bacteria associated with human diseases.

## Data Availability Statement

The RNA-Seq data generated and analyzed for this study have been deposited in the ArrayExpress database at EMBL-EBI (www.ebi.ac.uk/arrayexpress) under accession number E-MTAB-7138.

## Author Contributions

EC carried out most of the practical work (growth assays, DNA extraction, qPCR, RNA extraction, and genome mining) and data analysis. GLG performed the NMR analyses. NJ supervised the research at QIB. IM and HF helped with the analysis of *R. bromii* transcriptomics. EC and NJ wrote the manuscript with contributions from JL-G, IM, and HF.

## Conflict of Interest Statement

The authors declare that the research was conducted in the absence of any commercial or financial relationships that could be construed as a potential conflict of interest.The reviewer GG and handling Editor declared their shared affiliation.

## Footnotes

1 http://www-huber.embl.de/HTSeq/doc/count.html

2 http://www.hmdb.ca/

## Abbreviations

Padj: adjusted *p*-value
RS: resistant starch
SS: soluble starch.

## References

[B1] Almagro-Moreno S. Boyd E. F. (2009). Insights into the evolution of sialic acid catabolism among bacteria. *BMC Evol. Biol.* 9:118. 10.1186/1471-2148-9-118 19470179 PMC2693436

[B2] Belzer C. Chia L. W. Aalvink S. Chamlagain B. Piironen V. Knol J. (2017). Microbial metabolic networks at the mucus layer lead to diet-independent butyrate and vitamin B12 production by intestinal symbionts. *mBio* 8:e00770-17. 10.1128/mBio.00770-17 28928206 PMC5605934

[B3] Boratyn G. M. Camacho C. Cooper P. S. Coulouris G. Fong A. Ma N. (2013). BLAST: a more efficient report with usability improvements. *Nucleic Acids Res.* 41 W29–W33. 10.1093/nar/gkt282 23609542 PMC3692093

[B4] Bunesova V. Lacroix C. Schwab C. (2018). Mucin cross-feeding of infant Bifidobacteria and Eubacterium hallii. *Microb. Ecol.* 75 228–238. 10.1007/s00248-017-1037-4 28721502

[B5] Centanni M. Hutchison J. C. Carnachan S. M. Daines A. M. Kelly W. J. Tannock G. W. (2017). Differential growth of bowel commensal *Bacteroides* species on plant xylans of differing structural complexity. *Carbohydr. Polym.* 157 1374–1382. 10.1016/j.carbpol.2016.11.017 27987846

[B6] Centanni M. Lawley B. Butts C. A. Roy N. Lee J. Kelly W. J. (2018). Bifidobacterium pseudolongum has characteristics of a keystone species in bifidobacterial blooms in the ceca of rats fed Hi-Maize starch. *Appl. Environ. Microbiol.* 10.1128/AEM.00547-18 [Epub ahead of print]. 29802187 PMC6052269

[B7] Chia L. W. Hornung B. V. H. Aalvink S. Schaap P. J. de Vos W. M. Knol J. (2018). Deciphering the trophic interaction between *Akkermansia muciniphila* and the butyrogenic gut commensal *Anaerostipes caccae* using a metatranscriptomic approach. *Antonie Van Leeuwenhoek* 111 859–873. 10.1007/s10482-018-1040-x 29460206 PMC5945754

[B8] Crost E. H. Tailford L. E. Le Gall G. Fons M. Henrissat B. Juge N. (2013). Utilisation of mucin glycans by the human gut symbiont *Ruminococcus gnavus* is strain-dependent. *PLoS One* 8:e76341. 10.1371/journal.pone.0076341 24204617 PMC3808388

[B9] Crost E. H. Tailford L. E. Monestier M. Swarbreck D. Henrissat B. Crossman L. C. (2016). The mucin-degradation strategy of *Ruminococcus gnavus*: the importance of intramolecular trans-sialidases. *Gut Microbes* 7 302–312. 10.1080/19490976.2016.1186334 27223845 PMC4988440

[B10] David L. A. Maurice C. F. Carmody R. N. Gootenberg D. B. Button J. E. Wolfe B. E. (2014). Diet rapidly and reproducibly alters the human gut microbiome. *Nature* 505 559–563. 10.1038/nature12820 24336217 PMC3957428

[B11] Donaldson G. P. Lee S. M. Mazmanian S. K. (2016). Gut biogeography of the bacterial microbiota. *Nat. Rev. Microbiol.* 14 20–32. 10.1038/nrmicro3552 26499895 PMC4837114

[B12] Duncan S. H. Hold G. L. Barcenilla A. Stewart C. S. Flint H. J. (2002). *Roseburia intestinalis* sp. nov., a novel saccharolytic, butyrate-producing bacterium from human faeces. *Int. J. Syst. Evol. Microbiol.* 52(Pt 5), 1615–1620. 10.1099/00207713-52-5-1615 12361264

[B13] Flint H. J. Duncan S. H. Louis P. (2017). The impact of nutrition on intestinal bacterial communities. *Curr. Opin. Microbiol.* 38 59–65. 10.1016/j.mib.2017.04.005 28486162

[B14] Gunning A. P. Kirby A. R. Fuell C. Pin C. Tailford L. E. Juge N. (2013). Mining the “glycocode”–exploring the spatial distribution of glycans in gastrointestinal mucin using force spectroscopy. *FASEB J.* 27 2342–2354. 10.1096/fj.12-221416 23493619 PMC3659345

[B15] Hoek M. Merks R. M. H. (2017). Emergence of microbial diversity due to cross-feeding interactions in a spatial model of gut microbial metabolism. *BMC Syst. Biol.* 11:56. 10.1186/s12918-017-0430-4 28511646 PMC5434578

[B16] Koropatkin N. M. Cameron E. A. Martens E. C. (2012). How glycan metabolism shapes the human gut microbiota. *Nat. Rev. Microbiol.* 10 323–335. 10.1038/nrmicro2746 22491358 PMC4005082

[B17] Lawson P. A. Finegold S. M. (2015). Reclassification of *Ruminococcus obeum* as *Blautia obeum* comb. nov. *Int. J. Syst. Evol. Microbiol.* 65(Pt 3), 789–793. 10.1099/ijs.0.000015 25481290

[B18] Liu C. Finegold S. M. Song Y. Lawson P. A. (2008). Reclassification of *Clostridium coccoides*, *Ruminococcus hansenii*, *Ruminococcus hydrogenotrophicus*, *Ruminococcus luti*, *Ruminococcus productus* and *Ruminococcus schinkii* as *Blautia coccoides* gen. nov., comb. nov., *Blautia hansenii* comb. nov., *Blautia hydrogenotrophica* comb. nov., *Blautia luti* comb. nov., *Blautia producta* comb. nov., *Blautia schinkii* comb. nov. and description of *Blautia wexlerae* sp. nov., isolated from human faeces. *Int. J. Syst. Evol. Microbiol.* 58(Pt 8), 1896–1902. 10.1099/ijs.0.65208-0 18676476

[B19] Lombard V. Golaconda Ramulu H. Drula E. Coutinho P. M. Henrissat B. (2014). The carbohydrate-active enzymes database (CAZy) in 2013. *Nucleic Acids Res.* 42 D490–D495. 10.1093/nar/gkt1178 24270786 PMC3965031

[B20] Love M. I. Huber W. Anders S. (2014). Moderated estimation of fold change and dispersion for RNA-seq data with DESeq2. *Genome Biol.* 15:550. 10.1186/s13059-014-0550-8 25516281 PMC4302049

[B21] Mark Welch J. L. Hasegawa Y. McNulty N. P. Gordon J. I. Borisy G. G. (2017). Spatial organization of a model 15-member human gut microbiota established in gnotobiotic mice. *Proc. Natl. Acad. Sci. U.S.A.* 114 E9105–E9114. 10.1073/pnas.1711596114 29073107 PMC5664539

[B22] Metsalu T. Vilo J. (2015). ClustVis: a web tool for visualizing clustering of multivariate data using Principal Component Analysis and heatmap. *Nucleic Acids Res.* 43 W566–W570. 10.1093/nar/gkv468 25969447 PMC4489295

[B23] Milani C. Lugli G. A. Duranti S. Turroni F. Mancabelli L. Ferrario C. (2015). Bifidobacteria exhibit social behavior through carbohydrate resource sharing in the gut. *Sci. Rep.* 5:15782. 10.1038/srep15782 26506949 PMC4623478

[B24] Moore W. E. C. Johnson J. L. Holdeman L. V. (1976). Emendation of Bacteroidaceae and *Butyrivibrio* and descriptions of *Desulfomonas* gen. nov. and ten new species in the genera *Desulfomonas*, *Butyrivibrio*, *Eubacterium*, *Clostridium*, and *Ruminococcus*. *Int. J. Syst. Evol. Microbiol.* 26 238–252. 10.1099/00207713-26-2-238

[B25] Mukhopadhya I. Morais S. Laverde-Gomez J. Sheridan P. O. Walker A. W. Kelly W. (2018). Sporulation capability and amylosome conservation among diverse human colonic and rumen isolates of the keystone starch-degrader *Ruminococcus bromii*. *Environ. Microbiol.* 20 324–336. 10.1111/1462-2920.14000 29159997 PMC5814915

[B26] Murata T. Yamato I. Igarashi K. Kakinuma Y. (1996). Intracellular Na^+^ regulates transcription of the ntp operon encoding a vacuolar-type Na^+^-translocating ATPase in *Enterococcus hirae*. *J. Biol. Chem.* 271 23661–23666. 8798587 10.1074/jbc.271.39.23661

[B27] Ndeh D. Gilbert H. J. (2018). Biochemistry of complex glycan depolymerisation by the human gut microbiota. *FEMS Microbiol. Rev.* 42 146–164. 10.1093/femsre/fuy002 29325042

[B28] Owen C. D. Tailford L. E. Monaco S. Suligoj T. Vaux L. Lallement R. (2017). Unravelling the specificity and mechanism of sialic acid recognition by the gut symbiont *Ruminococcus gnavus*. *Nat. Commun.* 8:2196. 10.1038/s41467-017-02109-8 29259165 PMC5736709

[B29] Pereira F. C. Berry D. (2017). Microbial nutrient niches in the gut. *Environ. Microbiol.* 19 1366–1378. 10.1111/1462-2920.13659 28035742 PMC5412925

[B30] Qin J. Li R. Raes J. Arumugam M. Burgdorf K. S. Manichanh C. (2010). A human gut microbial gene catalogue established by metagenomic sequencing. *Nature* 464 59–65. 10.1038/nature08821 20203603 PMC3779803

[B31] Reichardt N. Duncan S. H. Young P. Belenguer A. McWilliam Leitch C. Scott K. P. (2014). Phylogenetic distribution of three pathways for propionate production within the human gut microbiota. *ISME J.* 8 1323–1335. 10.1038/ismej.2014.14 24553467 PMC4030238

[B32] Rogowski A. Briggs J. A. Mortimer J. C. Tryfona T. Terrapon N. Lowe E. C. (2015). Glycan complexity dictates microbial resource allocation in the large intestine. *Nat. Commun.* 6:7481. 10.1038/ncomms8481 26112186 PMC4491172

[B33] Sambrook J. Fritsch E. F. Maniatis T. (1989). *Molecular Cloning: A Laboratory Manual*. Cold Spring Harbor, NY: Cold Spring Harbor Laboratory.

[B34] Sonnenburg E. D. Smits S. A. Tikhonov M. Higginbottom S. K. Wingreen N. S. Sonnenburg J. L. (2016). Diet-induced extinctions in the gut microbiota compound over generations. *Nature* 529 212–215. 10.1038/nature16504 26762459 PMC4850918

[B35] Tailford L. E. Crost E. H. Kavanaugh D. Juge N. (2015a). Mucin glycan foraging in the human gut microbiome. *Front. Genet.* 6:81. 10.3389/fgene.2015.00081 25852737 PMC4365749

[B36] Tailford L. E. Owen C. D. Walshaw J. Crost E. H. Hardy-Goddard J. Le Gall G. (2015b). Discovery of intramolecular trans-sialidases in human gut microbiota suggests novel mechanisms of mucosal adaptation. *Nat. Commun.* 6:7624. 10.1038/ncomms8624 26154892 PMC4510645

[B37] Tannock G. W. Wilson C. M. Loach D. Cook G. M. Eason J. O’Toole P. W. (2012). Resource partitioning in relation to cohabitation of Lactobacillus species in the mouse forestomach. *ISME J.* 6 927–938. 10.1038/ismej.2011.161 22094343 PMC3329185

[B38] Thursby E. Juge N. (2017). Introduction to the human gut microbiota. *Biochem. J.* 474 1823–1836. 10.1042/BCJ20160510 28512250 PMC5433529

[B39] Tropini C. Earle K. A. Huang K. C. Sonnenburg J. L. (2017). The gut microbiome: connecting spatial organization to function. *Cell Host Microbe* 21 433–442. 10.1016/j.chom.2017.03.010 28407481 PMC5576359

[B40] Turroni F. Bottacini F. Foroni E. Mulder I. Kim J. H. Zomer A. (2010). Genome analysis of Bifidobacterium bifidum PRL2010 reveals metabolic pathways for host-derived glycan foraging. *Proc. Natl. Acad. Sci. U.S.A.* 107 19514–19519. 10.1073/pnas.1011100107 20974960 PMC2984195

[B41] Turroni F. Milani C. Duranti S. Mahony J. van Sinderen D. Ventura M. (2017). Glycan utilization and cross-feeding activities by Bifidobacteria. *Trends Microbiol.* 26 339–350. 10.1016/j.tim.2017.10.001 29089173

[B42] Turroni F. Strati F. Foroni E. Serafini F. Duranti S. van Sinderen D. (2012). Analysis of predicted carbohydrate transport systems encoded by *Bifidobacterium bifidum* PRL2010. *Appl. Environ. Microbiol.* 78 5002–5012. 10.1128/AEM.00629-12 22562993 PMC3416360

[B43] Williams B. B. Van Benschoten A. H. Cimermancic P. Donia M. S. Zimmermann M. Taketani M. (2014). Discovery and characterization of gut microbiota decarboxylases that can produce the neurotransmitter tryptamine. *Cell Host Microbe* 16 495–503. 10.1016/j.chom.2014.09.001 25263219 PMC4260654

[B44] Ze X. Ben David Y. Laverde-Gomez J. A. Dassa B. Sheridan P. O. Duncan S. H. (2015). Unique organization of extracellular amylases into amylosomes in the resistant starch-utilizing human colonic firmicutes bacterium *Ruminococcus bromii*. *mBio* 6:e01058-15. 10.1128/mBio.01058-15 26419877 PMC4611034

[B45] Ze X. Duncan S. H. Louis P. Flint H. J. (2012). *Ruminococcus bromii* is a keystone species for the degradation of resistant starch in the human colon. *ISME J.* 6 1535–1543. 10.1038/ismej.2012.4 22343308 PMC3400402

[B46] Ze X. Le Mougen F. Duncan S. H. Louis P. Flint H. J. (2013). Some are more equal than others: the role of “keystone” species in the degradation of recalcitrant substrates. *Gut Microbes* 4 236–240. 10.4161/gmic.23998 23549436 PMC3669169

