# Supplementary material for: Mechanistic Insights Into the Cross-Feeding of Ruminococcus gnavus and Ruminococcus bromii on Host and Dietary Carbohydrates
Source: Front Microbiol. 2018 Nov 5;9:2558. doi: 10.3389/fmicb.2018.02558 (PMC6231298; doi:10.3389/fmicb.2018.02558)
Supplement: Supplementary file 1 [file Presentation_1.pdf]

Table S1: Primers used to quantify bacteria by qPCR

Primer name	Sequence	Target (16S rRNA gene of:)	Amplicon size	Reference
UniF	GTGSTGCAYGGYYGTCGTCA	All bacteria	147-148nt	(Fuller et al., 2007)
UniR	ACGTCRTCCMCNCCTTCCTC			
Rflbr730F	GGCGGCYTRCTGGGCTTT	cluster IV *Ruminococcus* spp.	157nt	(Ramirez-Farias et al., 2009)
Clep866mR	CCAGGTGGATWACTTATTGTG TTAA			
16SRgF	TGGCGGCGTGCTTAACA	*Ruminococcus gnavus*	57nt	(Joossens et al., 2011)
16RgR	TCCGAAGAAATCCGTCAAGGT			

Fuller, Z., Louis, P., Mihajlovski, A., Rungapamestry, V., Ratcliffe, B., and Duncan, A.J. (2007). Influence of cabbage processing methods and prebiotic manipulation of colonic microflora on glucosinolate breakdown in man. *Br J Nutr* 98(2)**,** 364-372. doi: 10.1017/S0007114507709091.

Joossens, M., Huys, G., Cnockaert, M., De Preter, V., Verbeke, K., Rutgeerts, P., et al. (2011). Dysbiosis of the faecal microbiota in patients with Crohn's disease and their unaffected relatives. *Gut* 60(5)**,** 631-637. doi: 10.1136/gut.2010.223263.

Ramirez-Farias, C., Slezak, K., Fuller, Z., Duncan, A., Holtrop, G., and Louis, P. (2009). Effect of inulin on the human gut microbiota: stimulation of Bifidobacterium adolescentis and Faecalibacterium prausnitzii. *Br J Nutr* 101(4)**,** 541-550. doi: 10.1017/S0007114508019880.

Table S2: Sequences used as reference for genome mining

Target protein	Sequence used as reference:		NCBI reference sequence?
	Accession number	Organism	
PduQ	NP_460997.1	*Salmonella enterica* subsp. *enterica* serovar Typhimurium str. LT2	yes
PduC	NP_460985.1		yes
PduD	NP_460986.1		yes
PduE	NP_460987.1		yes
Lactaldehyde reductase	EDY33573.1	*Ruminococcus lactaris* ATCC 29176	no
PduP	SCY17815.1	*Blautia* sp. SF-50	no
acryloyl-CoA reductase	GAX63847.1	*Clostridioides difficile*	no
methylmalonyl-CoA carboxytransferase	ACJ24327.1	*Clostridium symbiosum*	no
FucU	NP_417284.1	*Escherichia coli* str. K-12 substr. MG1655	yes
FucI	NP_417282.1		yes
FucK	NP_417283.2		yes
L-fuculose-1-phosphate aldolase	NP_417280.1		yes

Table S3: List of differentially expressed *R. bromii* genes

Gene name:	Annotation	Rb_SS vs Rb+Rg_SS*		Rb_RS vs Rb+Rg_RS*		Rb_SS vs Rb_RS*		Rb+Rg_SS vs Rb+Rg_RS*	
		Log2FC**	padj	Log2FC	padj	Log2FC	padj	Log2FC	padj
L2-63_00114	gltB Ferredoxin-dependent glutamate synthase 1	1.41	1.26E-02	1.55	1.69E-03	0.11	9.79E-01	0.26	7.00E-01
L2-63_00390	serine/threonine protein kinase	-0.18	8.41E-01	-2.3	3.31E-10	1.02	2.59E-01	-1.1	2.06E-02
L2-63_00637	trpE Anthranilate synthase component 1	-3.22	1.42E-26	-3.09	7.42E-24	-0.36	8.47E-01	-0.23	6.29E-01
L2-63_00638	trpG Anthranilate synthase component II	-3	3.16E-23	-2.94	9.62E-22	-0.29	9.19E-01	-0.23	6.33E-01
L2-63_00639	trpD2 Anthranilate phosphoribosyltransferase 2	-3.12	1.42E-26	-2.78	8.61E-21	-0.43	7.52E-01	-0.09	8.50E-01
L2-63_00640	trpC Indole-3-glycerol phosphate synthase	-3.09	1.72E-24	-2.7	2.49E-18	-0.42	7.89E-01	-0.03	9.58E-01
L2-63_00641	trpB_2 Tryptophan synthase beta chain	-3.22	7.35E-28	-2.43	1.44E-15	-0.56	6.54E-01	0.23	6.31E-01
L2-63_00642	trpA Tryptophan synthase alpha chain	-3.23	2.06E-28	-2.37	4.53E-15	-0.53	6.61E-01	0.34	4.39E-01
L2-63_00643	cobB NAD-dependent deacetylase	-3.3	2.71E-29	-2.22	2.98E-13	-0.64	6.03E-01	0.44	3.06E-01
L2-63_00722	metH B12-dependent methionine synthase	-0.16	8.15E-01	1.53	4.71E-07	-0.56	6.43E-01	1.13	1.06E-03
L2-63_00870	ydjE Inner membrane metabolite transport protein	0.29	7.65E-01	-1.66	9.62E-04	0.78	6.54E-01	-1.17	4.12E-02
L2-63_00871	gutB Sorbitol dehydrogenase	0.12	9.19E-01	-1.91	6.39E-05	0.76	6.54E-01	-1.26	2.53E-02
L2-63_00872	gatY D-tagatose-1,6-bisphosphate aldolase subunit	0.14	8.92E-01	-2.24	4.97E-07	0.82	6.43E-01	-1.56	3.06E-03
L2-63_00873	ydjH Uncharacterized sugar kinase	0.14	8.94E-01	-2.27	3.73E-07	0.74	6.54E-01	-1.66	1.06E-03
L2-63_00874	yhdN General stress protein 69	0.05	9.66E-01	-2.54	4.92E-09	0.77	6.54E-01	-1.82	2.52E-04
L2-63_00875	glcR_2 HTH-type transcriptional repressor	-0.19	7.68E-01	-1.69	3.54E-08	0.25	9.33E-01	-1.25	2.82E-04
L2-63_00876	gltP Glutamate-aspartate carrier protein	-0.02	9.91E-01	-1.61	2.57E-05	0.58	6.69E-01	-1.02	2.70E-02
L2-63_00933	fus_1 Elongation factor G	-0.62	3.87E-01	-2.06	8.71E-06	0.28	9.45E-01	-1.16	4.03E-02
L2-63_01124	adhE_2 Aldehyde-alcohol dehydrogenase	-0.4	5.81E-01	-2.33	4.92E-09	0.06	9.84E-01	-1.88	2.44E-05
L2-63_01219	ntpD_1 V-type sodium pump subunit D	0	9.99E-01	-1.61	3.79E-06	-0.13	9.69E-01	-1.74	3.25E-06
L2-63_01220	ntpB_1 V-type sodium pump subunit B	-0.01	9.92E-01	-1.68	1.19E-06	-0.15	9.60E-01	-1.82	1.29E-06
L2-63_01221	ntpA V-type sodium ATPase catalytic subunit A	0.05	9.62E-01	-1.66	1.80E-05	-0.14	9.69E-01	-1.85	4.03E-06
L2-63_01222	ntpG V-type sodium pump subunit G	-0.02	9.90E-01	-1.68	3.17E-05	0.05	9.84E-01	-1.62	2.19E-04
L2-63_01223	ntpC V-type sodium pump subunit C	0.2	8.15E-01	-1.57	6.39E-05	0.03	9.98E-01	-1.74	2.44E-05
L2-63_01224	V-type ATP synthase subunit E	0.13	8.83E-01	-1.59	3.17E-05	0.08	9.81E-01	-1.63	6.15E-05
L2-63_01225	ntpK Sodium ATPase proteolipid component	0.16	8.60E-01	-1.49	2.76E-04	0.03	9.98E-01	-1.61	1.85E-04
L2-63_01277	metK S-adenosylmethionine synthase	1.16	1.93E-02	2.65	5.01E-13	-0.64	6.61E-01	0.86	7.68E-02
L2-63_01983	metE 5-methyltetrahydropteroyltriglutamate	0.01	9.96E-01	1.62	4.19E-07	-0.34	8.65E-01	1.27	3.25E-04

*Condition A vs Condition B: genes with an up-regulated transcription in Condition A (Log2FC>1.5 and padj<0.05) are highlighted in red while those with an up-regulated transcription in Condition B (Log2FC<-1.5 and padj<0.05) are highlighted in green.
**Log2FC: Log2(Fold change in transcription level)

Table S4: List of differentially expressed *R. gnavus* genes

Gene name	Annotation	Rg_Glc vs Rb+Rg_SS*		Rg_Glc vs Rb+Rg_RS*		Rb+Rg_SS vs Rb+Rg_RS*	
		Log2FC**	padj	Log2FC**	padj	Log2FC**	padj
RUMGNA_00005	Hypothetical protein	-0.79	1.80E-01	1.06	1.10E-02	1.85	8.44E-07
RUMGNA_00006	DNA-binding protein	-0.73	1.77E-01	1.8	2.74E-07	2.54	2.89E-14
RUMGNA_00044	Putative integrase			1.98	1.57E-02	1.93	1.53E-02
RUMGNA_00047	Hypothetical protein	-0.51	7.15E-01	-1.55	2.16E-02	-1.05	1.30E-01
RUMGNA_00071	Hypothetical protein	-0.35	7.25E-01	1.63	6.48E-04	1.98	1.12E-05
RUMGNA_00095	Hypothetical protein	-0.83	4.70E-01	1.43	4.74E-02	2.26	4.01E-04
RUMGNA_00100	Hypothetical protein	0.01	9.95E-01	1.81	3.54E-08	1.8	5.21E-08
RUMGNA_00258	stage III sporulation protein AA	-0.05	9.80E-01	-1.52	1.93E-03	-1.48	2.63E-03
RUMGNA_00294	cation-translocating P-type ATPase	-0.88	1.04E-01	0.92	2.52E-02	1.8	6.09E-07
RUMGNA_00358	SPFH/Band 7/PHB domain protein	-0.23	8.41E-01	-1.55	1.68E-03	-1.32	5.52E-03
RUMGNA_00359	NfeD family protein	-0.36	7.87E-01	-1.8	3.41E-03	-1.44	1.58E-02
RUMGNA_00369	cold shock domain-containing protein	0.34	7.01E-01	1.98	1.12E-06	1.64	4.67E-05
RUMGNA_00385	Hypothetical protein	-1.01	8.46E-02	0.89	5.69E-02	1.9	2.90E-06
RUMGNA_00409	transcriptional regulator	-0.32	6.92E-01	2.11	1.56E-07	2.43	1.05E-09
RUMGNA_00464	MerR family transcriptional regulator	-0.66	3.51E-01	1.39	1.59E-03	2.05	4.09E-07
RUMGNA_00488	methionine adenosyltransferase			1.53	1.16E-01	1.97	2.74E-02
RUMGNA_00492	Hypothetical protein	1.22	2.41E-01	-1.24	5.59E-02	-2.45	1.31E-04
RUMGNA_00508	Hypothetical protein	-0.03	9.88E-01	1.77	7.51E-03	1.8	4.84E-03
RUMGNA_00509	ABC transporter ATP-binding protein			1.15	1.90E-01	1.78	2.36E-02
RUMGNA_00510	ABC transporter permease	-0.02	9.88E-01	1.54	1.64E-03	1.56	1.12E-03
RUMGNA_00511	ABC transporter permease	-0.22	8.37E-01	1.38	5.20E-03	1.6	7.45E-04
RUMGNA_00514	DNA-binding response regulator			1.5	6.78E-02	1.62	3.92E-02
RUMGNA_00525	putative hydroxydechloroatrazine ethylaminohydrolase	0.71	6.17E-01	2.18	4.92E-03	1.47	6.29E-02
RUMGNA_00529	NADH oxidase	0.43	5.69E-01	-1.11	7.04E-03	-1.54	4.42E-05
RUMGNA_00530	AraC family transcriptional regulator	0.24	8.05E-01	-1.28	3.28E-03	-1.51	2.21E-04
RUMGNA_00532	Hypothetical protein	0.46	5.98E-01	-1.72	5.35E-05	-2.18	1.49E-07
RUMGNA_00539	Hypothetical protein	-0.7	4.94E-01	1.73	2.95E-03	2.43	5.67E-06
RUMGNA_00542	HTH domain-containing protein; TrmB family transcriptional regulator	-1.12	5.54E-05	0.8	4.41E-03	1.93	5.70E-15
RUMGNA_00585	2-isopropylmalate synthase	-1.45	1.18E-04	0.19	7.00E-01	1.64	1.79E-06
RUMGNA_00610	Hypothetical protein	-0.1	9.11E-01	2.43	1.84E-16	2.53	8.08E-18
RUMGNA_00611	Hypothetical protein	0.02	9.88E-01	2.5	2.73E-09	2.48	6.40E-09
RUMGNA_00612	Hypothetical protein	-1	2.16E-01	1.27	3.27E-02	2.27	1.64E-05
RUMGNA_00613	Hypothetical protein	-1.28	4.10E-02	1.35	1.49E-02	2.63	8.71E-08
RUMGNA_00614	50S ribosomal protein L20	-0.9	1.59E-01	0.87	6.91E-02	1.77	1.90E-05
RUMGNA_00615	50S ribosomal protein L35	-0.77	3.14E-01	1.04	4.51E-02	1.81	7.21E-05

RUMGNA_00616	translation initiation factor IF-3	-0.77	3.92E-01	1.22	3.27E-02	1.99	7.90E-05
RUMGNA_00651	Hypothetical protein	-0.54	5.90E-01	1.07	5.85E-02	1.61	1.39E-03
RUMGNA_00652	XRE family transcriptional regulator	-0.76	2.72E-01	1.89	2.12E-05	2.65	2.41E-10
RUMGNA_00653	Hypothetical protein	0.21	8.62E-01	2.39	2.19E-07	2.18	1.68E-06
RUMGNA_00655	RNA polymerase factor sigma-70	-0.61	2.67E-01	1.43	4.23E-05	2.04	4.79E-10
RUMGNA_00656	23S rRNA (guanosine(2251)-2'-O)-methyltransferase RlmB	-0.51	1.96E-01	1.06	4.88E-05	1.56	1.38E-10
RUMGNA_00666	potassium transporter	-0.9	3.10E-01	1.32	2.68E-02	2.22	2.69E-05
RUMGNA_00670	Hypothetical protein	-0.49	5.69E-01	1.32	6.38E-03	1.81	5.80E-05
RUMGNA_00672	DNA-directed RNA polymerase sigma-70 factor	-0.32	8.32E-01	1.26	8.67E-02	1.58	2.24E-02
RUMGNA_00681	AICARFT/IMPCHase bienzyme; phosphoribosylaminoimidazolecarboxamide formyltransferase	1	5.62E-01	-1.14	2.78E-01	-2.14	1.70E-02
RUMGNA_00682	inosine monophosphate cyclohydrolase	0.89	6.12E-01	-1.04	3.31E-01	-1.94	3.39E-02
RUMGNA_00692	XRE family transcriptional regulator	0.91	1.50E-01	-1	2.84E-02	-1.91	2.93E-06
RUMGNA_00744	SNF2 family N-terminal domain protein; helicase	1.13	9.08E-02	-1.72	1.93E-04	-2.85	1.51E-10
RUMGNA_00747	DUF4316 domain-containing protein	-0.05	9.79E-01	1.54	7.96E-03	1.6	4.23E-03
RUMGNA_00789	Hypothetical protein	-1.21	7.53E-02	0.55	3.61E-01	1.76	2.59E-04
RUMGNA_00792	50S ribosomal protein L13	-0.81	1.98E-01	1.14	9.96E-03	1.95	9.73E-07
RUMGNA_00793	Hypothetical protein	-0.64	4.65E-01	2.58	4.24E-08	3.22	1.70E-12
RUMGNA_00823	Hypothetical protein	-0.45	7.35E-01	1.43	3.39E-02	1.87	2.12E-03
RUMGNA_00826	Uncharacterized phosphatase YwpJ or cof-like hydrolase or haloacid dehalogenase	0.51	3.30E-01	-1.08	1.05E-03	-1.59	2.04E-07
RUMGNA_00827	transcriptional antiterminator BglG	0.48	4.34E-01	-1.34	1.30E-04	-1.82	3.08E-08
RUMGNA_00828	beta-glucosidase/GH3	0.69	3.35E-01	-1.84	1.85E-05	-2.53	5.03E-10
RUMGNA_00829	PTS system sucrose-specific IIBC component	0.52	5.98E-01	-2.38	8.84E-07	-2.9	5.23E-10
RUMGNA_00830	Sucrose-6-phosphate hydrolase or beta-fructofuranosidase	-0.1	9.58E-01	-2.76	6.18E-06	-2.66	9.03E-06
RUMGNA_00832	xylulokinase	0.53	4.58E-01	-2	1.19E-07	-2.53	5.58E-12
RUMGNA_00833	fucose isomerase	0.49	5.24E-01	-2.33	1.02E-09	-2.81	6.62E-14
RUMGNA_00834	DUF4867 domain-containing protein	0.64	4.40E-01	-2.28	1.01E-07	-2.92	1.31E-11
RUMGNA_00842	Fucosidase GH95	2.31	2.71E-07	1.15	1.78E-02	-1.16	1.09E-02
RUMGNA_00854	DUF4430 domain-containing protein	-0.77	2.34E-01	0.84	7.20E-02	1.61	7.56E-05
RUMGNA_00856	Hypothetical protein	-0.71	2.93E-01	0.82	8.45E-02	1.53	2.13E-04
RUMGNA_00857	Hypothetical protein	-0.13	9.03E-01	1.97	1.74E-07	2.1	1.06E-08
RUMGNA_00865	Hypothetical protein	0.1	9.40E-01	-5.53	4.37E-41	-5.63	3.99E-41
RUMGNA_00873	50S ribosomal protein L34	-0.32	7.20E-01	1.23	6.01E-03	1.55	1.54E-04
RUMGNA_00874	ribonuclease P protein component	-0.65	3.14E-01	1.16	6.42E-03	1.81	2.62E-06
RUMGNA_00875	membrane protein insertion efficiency factor YidD	-0.4	6.23E-01	1.35	1.33E-03	1.74	7.97E-06
RUMGNA_00876	membrane protein insertase YidC	-0.5	4.64E-01	1.03	9.21E-03	1.53	1.99E-05

RUMGNA_00882	Hypothetical protein	-0.22	8.58E-01	1.47	6.13E-03	1.69	8.86E-04
RUMGNA_00898	manganese efflux pump	-0.84	1.78E-01	0.83	7.37E-02	1.67	4.20E-05
RUMGNA_00904	Hypothetical protein	-0.89	2.09E-01	1.4	1.02E-02	2.29	4.09E-06
RUMGNA_00954	glutamine synthetase	-0.38	7.02E-01	-1.56	1.24E-03	-1.18	1.25E-02
RUMGNA_00958	transporter, major facilitator family protein or MFS transporter	-2.35	1.06E-08	-2.24	2.88E-08	0.11	8.42E-01
RUMGNA_00972	DNA-binding response regulator	0.5	7.05E-01	-1.09	9.92E-02	-1.59	1.11E-02
RUMGNA_00974	Hypothetical protein	0.71	1.64E-01	-1.68	2.07E-07	-2.39	1.96E-14
RUMGNA_00984	Hypothetical protein	-0.92	3.99E-01	0.75	3.43E-01	1.66	1.23E-02
RUMGNA_01004	AraC family transcriptional regulator	0.56	3.55E-01	-1.4	1.08E-04	-1.96	9.02E-09
RUMGNA_01006	rhamnulokinase	0.83	1.11E-01	-2.71	5.96E-19	-3.54	2.72E-28
RUMGNA_01007	putative L-rhamnose isomerase	0.38	5.46E-01	-2.3	2.76E-15	-2.68	1.85E-19
RUMGNA_01009	DUF4968 domain-containing protein	1.35	1.31E-02	-0.17	7.79E-01	-1.53	5.99E-04
RUMGNA_01017	glycyl radical protein	0.56	1.93E-01	-2.08	9.68E-16	-2.64	4.70E-25
RUMGNA_01018	glycyl-radical enzyme activating protein	0.77	1.97E-01	-1.48	1.78E-04	-2.25	1.39E-09
RUMGNA_01019	glycyl-radical enzyme activating protein	0.82	1.88E-01	-1.71	1.06E-05	-2.53	3.42E-11
RUMGNA_01036	AraC family transcriptional regulator	1.31	1.11E-02	-0.38	4.77E-01	-1.69	5.35E-05
RUMGNA_01045	mannonate dehydratase	0.98	6.68E-02	-1.4	1.36E-04	-2.38	6.62E-11
RUMGNA_01046	mannitol dehydrogenase family protein	0.67	2.16E-01	-1.45	1.69E-05	-2.12	1.37E-10
RUMGNA_01049	2-dehydro-3-deoxyphosphogluconate aldolase/4-hydroxy-2 oxoglutarate aldolase	0.45	5.63E-01	-1.52	1.78E-04	-1.97	2.33E-07
RUMGNA_01050	sugar kinase	0.56	4.45E-01	-1.17	7.02E-03	-1.73	1.20E-05
RUMGNA_01063	ABC transporter permease protein	2.51	3.41E-05	-0.16	8.44E-01	-2.67	1.51E-06
RUMGNA_01064	sugar ABC transporter ATP-binding protein	2.41	1.15E-04	0.02	9.86E-01	-2.4	2.69E-05
RUMGNA_01065	ABC transporter permease protein	2.43	3.11E-04	0.32	7.06E-01	-2.11	5.99E-04
RUMGNA_01066	LacI family transcriptional regulator	2.56	1.52E-06	-0.26	7.17E-01	-2.82	1.47E-08
RUMGNA_01067	Hypothetical protein	2.58	2.52E-07	0.61	3.17E-01	-1.98	4.68E-05
RUMGNA_01069	L-fuculokinase	1.36	4.69E-03	-0.92	4.56E-02	-2.28	7.73E-09
RUMGNA_01070	L-fucose isomerase	1.37	1.76E-04	-1.01	5.45E-03	-2.38	7.23E-14
RUMGNA_01071	L-fucose mutarotase	1.55	6.00E-05	-0.63	1.23E-01	-2.19	2.52E-10
RUMGNA_01085	NAD(P)-dependent alcohol dehydrogenase	1.94	1.18E-04	0.71	2.05E-01	-1.23	1.06E-02
RUMGNA_01086	GTP-binding protein	1.59	9.90E-04	0.46	4.02E-01	-1.14	1.05E-02
RUMGNA_01106	Cna B-type domain-containing protein	0.97	3.32E-02	-0.92	1.69E-02	-1.89	2.26E-08
RUMGNA_01115	50S ribosomal protein L19	-0.69	3.15E-01	1.08	1.82E-02	1.78	1.55E-05
RUMGNA_01116	tRNA (guanosine(37)-N1)-methyltransferase TrmD	-1.34	4.48E-02	1.01	7.59E-02	2.35	1.74E-06
RUMGNA_01117	16S rRNA processing protein RimM	-1.26	1.13E-01	1.01	1.04E-01	2.26	1.97E-05
RUMGNA_01118	KH domain-containing protein	-0.82	2.71E-01	0.86	1.07E-01	1.68	2.57E-04
RUMGNA_01119	30S ribosomal protein S16	-0.85	2.33E-01	0.82	1.14E-01	1.67	1.82E-04
RUMGNA_01120	signal recognition particle protein	-0.84	4.14E-01	0.98	1.48E-01	1.82	1.80E-03
RUMGNA_01121	DNA-binding protein	-0.77	4.05E-01	1.6	4.60E-03	2.37	3.97E-06

RUMGNA_01165	Abi family protein	-0.2	8.44E-01	1.48	7.29E-04	1.68	4.67E-05
RUMGNA_01171	Hypothetical protein	-1.03	1.67E-01	0.79	1.99E-01	1.83	4.70E-04
RUMGNA_01178	Hypothetical protein	0.81	3.37E-01	-0.78	1.47E-01	-1.6	1.70E-03
RUMGNA_01183	prepilin peptidase	0.88	7.92E-02	-1.16	1.49E-03	-2.04	1.73E-09
RUMGNA_01184	PHP domain protein	0.34	6.49E-01	-1.67	2.00E-06	-2.01	4.58E-09
RUMGNA_01186	cob(I)yrinic acid a,c-diamide adenosyltransferase	0.52	4.40E-01	-1.36	2.89E-04	-1.88	1.26E-07
RUMGNA_01187	glycerol dehydratase	0.59	3.92E-01	-1.18	2.78E-03	-1.77	3.00E-06
RUMGNA_01188	diol dehydratase reactivase subunit alpha	0.54	3.97E-01	-1.66	3.96E-06	-2.2	1.57E-10
RUMGNA_01189	propanediol dehydratase small subunit PduE	0.6	4.56E-01	-1.46	7.89E-04	-2.06	1.16E-06
RUMGNA_01190	propanediol dehydratase medium subunit PduD	0.58	4.50E-01	-1.23	5.53E-03	-1.81	1.12E-05
RUMGNA_01191	propanediol dehydratase large subunit PduC	0.59	2.79E-01	-1.55	4.23E-06	-2.15	2.62E-11
RUMGNA_01193	Hypothetical protein	0.03	9.74E-01	1.7	1.83E-08	1.67	3.16E-08
RUMGNA_01194	Na+/H+ antiporter family protein	0.28	7.54E-01	1.82	6.84E-06	1.54	1.05E-04
RUMGNA_01195	bifunctional hydroxymethylpyrimidine kinase/phosphomethylpyrimidine kinase	1.59	1.77E-02	2.14	9.65E-05	0.55	3.95E-01
RUMGNA_01196	HAD family phosphatase	2.27	7.80E-04	2.57	2.43E-05	0.3	7.19E-01
RUMGNA_01197	thiamine phosphate synthase	2.05	1.19E-04	2.24	3.80E-06	0.2	7.83E-01
RUMGNA_01198	hydroxyethylthiazole kinase	1.82	1.48E-03	1.97	1.31E-04	0.15	8.34E-01
RUMGNA_01199	Hypothetical protein	1.85	4.64E-02	1.6	3.22E-02	-0.25	8.02E-01
RUMGNA_01214	1,3-beta-galactosyl-N-acetylhexosamine phosphorylase	0.47	4.64E-01	-1.13	1.64E-03	-1.6	1.81E-06
RUMGNA_01278	30S ribosomal protein S6	-0.85	1.42E-01	1.05	1.25E-02	1.91	3.74E-07
RUMGNA_01279	ingle-stranded DNA-binding protein	-0.78	1.94E-01	0.94	2.86E-02	1.72	5.85E-06
RUMGNA_01280	30S ribosomal protein S18	-0.84	1.49E-01	0.88	4.45E-02	1.72	5.81E-06
RUMGNA_01318	ABC transporter permease	2.05	5.84E-05	1.91	9.69E-05	-0.14	8.39E-01
RUMGNA_01319	ABC transporter ATP-binding protein	2.32	2.47E-05	2.27	1.53E-05	-0.05	9.45E-01
RUMGNA_01320	TetR/AcrR family transcriptional regulator	2.08	1.55E-04	2.27	1.09E-05	0.18	7.93E-01
RUMGNA_01329	Hypothetical protein	-0.35	7.05E-01	1.48	2.37E-03	1.83	8.74E-05
RUMGNA_01366	HPr family phosphocarrier protein	0.21	7.78E-01	1.5	2.25E-06	1.29	3.51E-05
RUMGNA_01367	Hypothetical protein	-0.52	5.77E-01	1.61	2.75E-03	2.13	2.75E-05
RUMGNA_01382	Hypothetical protein	0.1	9.42E-01	-1.63	1.01E-03	-1.74	4.46E-04
RUMGNA_01409	Hypothetical protein			0.81	4.27E-01	2.05	1.41E-02
RUMGNA_01460	ABC transporter ATP-binding protein	-0.68	5.65E-01	0.85	2.62E-01	1.53	2.22E-02
RUMGNA_01469	plantaricin C family lantibiotic	0.16	8.85E-01	-1.62	7.84E-05	-1.78	8.86E-06
RUMGNA_01490	signal peptidase I	0.78	3.39E-01	-0.95	7.31E-02	-1.73	3.10E-04
RUMGNA_01492	Rrf2 family transcriptional regulator	0.33	7.20E-01	-1.39	1.71E-03	-1.73	3.48E-05
RUMGNA_01493	LPXTG-motif cell wall anchor domain protein; Cna B-type domain-containing protein	0.79	4.89E-01	-1.24	7.30E-02	-2.03	8.76E-04
RUMGNA_01496	Hypothetical protein	1.13	1.97E-01	-0.49	5.05E-01	-1.61	5.46E-03
RUMGNA_01505	Hypothetical protein	-0.47	6.04E-01	1.35	5.82E-03	1.82	5.77E-05
RUMGNA_01506	aldehyde dehydrogenase (NAD) family protein	-0.4	6.29E-01	1.22	6.08E-03	1.62	6.77E-05

RUMGNA_01529	Hypothetical protein	0.63	2.55E-01	-1.37	5.95E-05	-2	1.03E-09
RUMGNA_01543	AAA family ATPase	0.19	7.49E-01	-1.34	1.93E-07	-1.53	1.00E-09
RUMGNA_01568	Hypothetical protein	0.09	9.54E-01	2.17	7.22E-05	2.08	7.90E-05
RUMGNA_01573	sodium-translocating pyrophosphatase	-2.01	2.89E-05	-0.01	9.92E-01	2.01	6.17E-06
RUMGNA_01579	glucose-1-phosphate adenylyltransferase	1.54	3.46E-05	-1.34	1.95E-04	-2.88	7.15E-19
RUMGNA_01580	glucose-1-phosphate adenylyltransferase subunit GlgD	1.4	9.53E-03	-1.09	2.84E-02	-2.48	7.10E-09
RUMGNA_01581	stage V sporulation protein G	1.39	3.32E-02	-1.11	4.88E-02	-2.5	2.73E-07
RUMGNA_01601	sugar ABC transporter substrate-binding protein	0.59	3.16E-01	-1.79	3.76E-07	-2.38	1.94E-12
RUMGNA_01602	sugar ABC transporter permease	0.65	2.67E-01	-0.98	1.13E-02	-1.63	4.79E-06
RUMGNA_01603	sugar ABC transporter permease	0.63	2.27E-01	-1.09	1.31E-03	-1.72	7.08E-08
RUMGNA_01611	Hypothetical protein	0.67	2.80E-01	-0.94	2.60E-02	-1.62	1.94E-05
RUMGNA_01622	Hypothetical protein	-1.23	1.97E-01	1.85	1.28E-02	3.08	4.18E-06
RUMGNA_01646	ketoacyl-ACP synthase III	-0.62	5.78E-01	2.77	1.09E-06	3.39	5.28E-10
RUMGNA_01647	acyl carrier protein	-0.95	2.49E-01	1.9	1.15E-03	2.86	1.21E-07
RUMGNA_01648	enoyl-[acyl-carrier-protein] reductase FabK	0.26	8.69E-01	2.53	5.39E-05	2.27	1.88E-04
RUMGNA_01649	[acyl-carrier-protein] S-malonyltransferase	-0.95	1.82E-01	2.32	5.09E-07	3.27	9.27E-14
RUMGNA_01650	3-oxoacyl-[acyl-carrier-protein] reductase	-1.08	1.61E-01	2.77	3.24E-08	3.86	9.39E-16
RUMGNA_01651	beta-ketoacyl-[acyl-carrier-protein] synthase II	-0.57	5.16E-01	2.65	3.63E-09	3.21	1.63E-13
RUMGNA_01652	putative acetyl-CoA carboxylase biotin carboxyl carrier protein subunit	-0.75	2.66E-01	3.05	2.19E-14	3.79	4.51E-22
RUMGNA_01653	3-hydroxyacyl-[acyl-carrier-protein] dehydratase FabZ	-0.76	2.27E-01	2.88	7.34E-14	3.64	5.75E-22
RUMGNA_01654	acetyl-CoA carboxylase biotin carboxylase subunit	-0.59	2.64E-01	2.77	1.32E-19	3.36	9.61E-29
RUMGNA_01655	acetyl-CoA carboxylase carboxyl transferase subunit	-0.63	2.38E-01	2.66	3.40E-17	3.28	3.82E-26
RUMGNA_01656	nitronate monooxygenase	-0.61	2.79E-01	2.78	2.81E-17	3.39	1.36E-25
RUMGNA_01657	MarR family transcriptional regulator	-0.58	3.35E-01	2.17	4.44E-10	2.75	4.22E-16
RUMGNA_01661	putative transposase	-2.02	1.81E-03	-0.41	5.93E-01	1.61	6.33E-03
RUMGNA_01662	putative DNA-binding transcriptional regulator CytR or LacI family transcriptional regulator	-2.02	2.57E-08	-1.02	9.42E-03	1	6.99E-03
RUMGNA_01664	Maltose/maltodextrin-binding protein precursor	-5.11	9.13E-43	-4.67	1.22E-35	0.44	3.56E-01
RUMGNA_01666	Maltose transport system permease protein malF	-5.36	3.97E-45	-4.93	4.16E-38	0.43	3.81E-01
RUMGNA_01667	sugar ABC transporter permease or arabinogalactan oligomer / maltooligosaccharide transport system permease protein	-5.35	9.76E-49	-5.02	1.19E-42	0.34	4.84E-01
RUMGNA_01668	type I pullulanase	-5.35	9.76E-49	-5.18	1.74E-45	0.17	7.53E-01
RUMGNA_01669	glycogen debranching protein or amylo-alpha-1,6-glucosidase	-4.83	1.65E-51	-4.26	4.90E-40	0.57	1.43E-01
RUMGNA_01670	maltose phosphorylase	-4.15	2.95E-37	-3.25	9.16E-23	0.9	1.50E-02
RUMGNA_01671	4-alpha-glucanotransferase	-4.4	5.40E-35	-3.96	3.29E-28	0.44	3.29E-01
RUMGNA_01672	alpha-glycosidase	-4.55	8.23E-40	-3.86	1.60E-28	0.69	9.13E-02
RUMGNA_01673	glucohydrolase or glucan 1,6-alpha-glucosidase	-4.59	9.84E-67	-3.84	2.25E-46	0.75	1.38E-02

RUMGNA_01679	MBL fold metallo-hydrolase	0.38	7.05E-01	-3.02	1.88E-11	-3.4	1.37E-14
RUMGNA_01680	Hypothetical protein	0.18	9.28E-01	-1.51	3.45E-02	-1.69	1.60E-02
RUMGNA_01682	RNA polymerase sporulation sigma factor SigG	0.23	8.77E-01	-2.06	2.45E-04	-2.29	3.29E-05
RUMGNA_01690	AarF/ABC1/UbiB kinase family protein	0.91	2.91E-02	-1.58	8.56E-07	-2.5	2.40E-16
RUMGNA_01692	AarF/ABC1/UbiB kinase family protein	1.17	7.80E-04	-1.6	3.73E-08	-2.76	7.38E-22
RUMGNA_01693	Hypothetical protein	1.34	2.35E-04	-2.41	9.89E-15	-3.76	3.13E-34
RUMGNA_01695	MoxR family ATPase	0.96	1.80E-01	-1.35	4.51E-03	-2.32	3.15E-07
RUMGNA_01696	DUF58 domain-containing protein	0.71	4.74E-01	-1.44	1.01E-02	-2.15	3.67E-05
RUMGNA_01697	transglutaminase domain-containing protein	0.83	1.94E-01	-1.58	1.82E-04	-2.41	9.63E-10
RUMGNA_01698	Hypothetical protein	0.58	6.43E-01	-1.11	7.86E-02	-1.69	5.78E-03
RUMGNA_01772	Glutamate-aspartate carrier protein or dicarboxylate/amino acid:cation symporter	1.98	1.19E-04	2.79	1.78E-09	0.81	1.33E-01
RUMGNA_01773	O-acetylhomoserine aminocarboxypropyltransferase/cysteine synthase	2.06	8.46E-07	2.44	6.00E-10	0.37	4.52E-01
RUMGNA_01774	pyridoxal 5'-phosphate synthase glutaminase subunit PdxT	1.87	1.58E-04	2	1.26E-05	0.13	8.42E-01
RUMGNA_01775	pyridoxal 5'-phosphate synthase lyase subunit PdxS	1.9	6.51E-03	1.76	4.99E-03	-0.14	8.68E-01
RUMGNA_01777	ATP synthase F1 subunit epsilon	-1.59	1.86E-02	-0.93	1.41E-01	0.66	2.88E-01
RUMGNA_01778	F0F1 ATP synthase subunit beta	-1.56	1.84E-02	-0.96	1.18E-01	0.61	3.27E-01
RUMGNA_01779	ATP synthase F1 subunit gamma	-1.54	1.76E-02	-0.95	1.14E-01	0.59	3.28E-01
RUMGNA_01780	F0F1 ATP synthase subunit alpha	-1.64	7.26E-03	-1.1	5.92E-02	0.55	3.64E-01
RUMGNA_01781	ATP synthase F1 subunit delta	-1.81	2.71E-03	-1.14	5.51E-02	0.67	2.67E-01
RUMGNA_01782	ATP synthase F0, B subunit	-1.97	1.29E-03	-1.2	5.00E-02	0.77	2.08E-01
RUMGNA_01783	ATP synthase F0 subunit C	-2.23	3.09E-04	-1.14	7.55E-02	1.09	7.01E-02
RUMGNA_01784	F0F1 ATP synthase subunit A	-2.14	3.26E-04	-1.27	3.81E-02	0.88	1.44E-01
RUMGNA_01785	Hypothetical protein	-1.72	3.34E-03	-1.1	5.49E-02	0.62	2.88E-01
RUMGNA_01786	Hypothetical protein			1.31	1.59E-01	1.78	3.79E-02
RUMGNA_01789	Hypothetical protein	0.34	7.72E-01	1.63	2.87E-03	1.29	1.79E-02
RUMGNA_01798	glucose-1-phosphate adenylyltransferase	1.92	1.26E-06	-0.94	2.68E-02	-2.87	2.58E-15
RUMGNA_01799	glucose-1-phosphate adenylyltransferase subunit GlgD	1.72	5.33E-06	-0.97	1.40E-02	-2.68	2.60E-15
RUMGNA_01807	GH2 glycoside hydrolase	0.55	6.24E-01	1.54	1.06E-02	0.99	1.13E-01
RUMGNA_01811	GH13 glycoside hydrolase/1,4-alpha-glucan branching protein GlgB	1.11	2.20E-03	-0.53	1.51E-01	-1.64	1.00E-07
RUMGNA_01878	Hypothetical protein	0.58	4.20E-01	1.89	2.59E-06	1.31	1.26E-03
RUMGNA_01883	Hypothetical protein	-0.61	4.05E-01	1.9	1.75E-05	2.51	3.01E-09
RUMGNA_01912	cytidylate kinase-like family protein	0.42	4.98E-01	-1.95	4.44E-10	-2.36	8.67E-15
RUMGNA_01925	ribosome silencing factor	-0.71	2.55E-01	0.81	6.67E-02	1.52	7.82E-05
RUMGNA_01926	HD domain-containing protein	-0.83	1.97E-01	1.05	2.30E-02	1.88	4.40E-06
RUMGNA_01927	nicotinate-nucleotide adenylyltransferase	-0.63	3.99E-01	1.14	1.40E-02	1.77	2.18E-05
RUMGNA_01928	ribosome assembly RNA-binding protein YhbY	-0.53	5.42E-01	1.31	6.40E-03	1.84	2.78E-05

RUMGNA_01930	Obg family GTPase CgtA	-0.63	3.93E-01	1.22	7.02E-03	1.85	6.56E-06
RUMGNA_01933	50S ribosomal protein L21	-0.81	1.59E-01	0.78	6.65E-02	1.59	1.73E-05
RUMGNA_01956	DegV family protein	-0.24	8.08E-01	2.51	1.41E-09	2.75	1.48E-11
RUMGNA_01978	ferredoxin	-0.23	8.12E-01	-1.51	3.53E-04	-1.28	1.92E-03
RUMGNA_01998	PTS system, glucose-like IIB component	0.33	8.72E-01	-1.75	4.77E-02	-2.07	9.94E-03
RUMGNA_02005	Hypothetical protein	-0.78	6.12E-01	1.02	2.82E-01	1.81	2.82E-02
RUMGNA_02029	RNA polymerase sporulation sigma factor SigK	-1.35	4.22E-02	0.92	1.34E-01	2.27	1.31E-05
RUMGNA_02030	ATP-binding protein	-0.35	8.00E-01	1.82	9.88E-03	2.17	1.08E-03
RUMGNA_02038	Hypothetical protein	0.35	8.05E-01	-1.87	1.30E-03	-2.22	1.83E-04
RUMGNA_02082	diversity-generating retroelement protein Avd	-0.75	6.49E-01	-2.26	5.03E-03	-1.51	6.37E-02
RUMGNA_02126	30S ribosomal protein S2	-0.65	3.92E-01	1	3.83E-02	1.65	1.02E-04
RUMGNA_02136	Hypothetical protein	0.63	5.37E-01	1.51	7.69E-03	0.88	1.21E-01
RUMGNA_02137	Hypothetical protein	0.61	5.22E-01	1.51	5.03E-03	0.9	9.68E-02
RUMGNA_02152	PTS beta-glucoside transporter subunit IIBCA	1.11	1.79E-01	-1.66	5.42E-04	-2.77	5.57E-08
RUMGNA_02153	PTS beta-glucoside transporter subunit IIBCA	1.01	7.30E-02	-1.07	8.58E-03	-2.09	8.01E-08
RUMGNA_02158	knotted carbamoyltransferase YgeW	1.27	1.56E-03	-2.67	1.84E-16	-3.93	8.46E-34
RUMGNA_02159	putative selenium metabolism hydrolase	1.22	1.20E-03	-2.62	1.02E-17	-3.84	7.08E-36
RUMGNA_02160	PLP-dependent lyase/thiolase or diaminopropionate ammonia-lyase	1.32	2.44E-03	-2.75	1.38E-15	-4.07	1.32E-31
RUMGNA_02161	YheO-like protein	1.4	1.13E-03	-2.72	7.70E-16	-4.13	4.73E-32
RUMGNA_02162	putative chlorohydrolase/aminohydrolase	1.87	1.34E-05	-1.92	3.08E-07	-3.79	4.69E-24
RUMGNA_02163	putative selenate reductase subunit YgfK	1.51	1.41E-02	-1.94	1.30E-04	-3.45	2.07E-13
RUMGNA_02165	molybdenum hydroxylase	1.68	4.03E-02	-0.48	5.39E-01	-2.16	4.97E-04
RUMGNA_02166	selenium-dependent xanthine dehydrogenase	1.18	3.07E-03	-1.04	4.66E-03	-2.22	1.34E-11
RUMGNA_02167	molybdate ABC transporter substrate-binding protein	-0.49	6.72E-01	-2.63	1.12E-06	-2.15	6.27E-05
RUMGNA_02168	molybdenum ABC transporter permease subunit	-0.56	6.46E-01	-1.93	2.01E-03	-1.36	2.66E-02
RUMGNA_02169	ABC transporter or Fe(3+) ions import ATP-binding protein FbpC 2	-0.74	4.78E-01	-1.86	1.55E-03	-1.13	5.68E-02
RUMGNA_02170	molybdenum transporter	-0.71	5.15E-01	-1.69	6.40E-03	-0.97	1.21E-01
RUMGNA_02229	phage tail family protein	1.27	3.18E-01	-0.52	5.84E-01	-1.79	2.68E-02
RUMGNA_02238	ECF transporter S component	1.35	7.27E-03	3.25	3.43E-16	1.89	5.39E-06
RUMGNA_02239	DEAD/DEAH box helicase	-1.22	3.43E-02	1.48	1.64E-03	2.69	1.77E-10
RUMGNA_02244	DNA-binding response regulator	0.53	2.65E-01	-1.03	4.99E-04	-1.56	4.55E-08
RUMGNA_02247	Hypothetical protein	-0.55	6.12E-01	1.04	8.29E-02	1.59	3.26E-03
RUMGNA_02251	Hypothetical protein	-0.73	4.30E-01	2.42	3.27E-06	3.15	1.79E-10
RUMGNA_02274	class B sortase	-0.44	7.17E-01	-1.82	1.04E-03	-1.38	1.41E-02
RUMGNA_02277	isopeptide-forming domain-containing fimbrial protein	-0.86	2.85E-01	-1.83	3.24E-04	-0.97	6.65E-02
RUMGNA_02278	LPXTG-motif cell wall anchor domain protein	-0.71	3.98E-01	-1.87	8.05E-05	-1.16	1.62E-02
RUMGNA_02309	Hypothetical protein	0.29	7.05E-01	1.73	1.90E-06	1.44	1.05E-04
RUMGNA_02324	phage major capsid protein	1.61	1.79E-03	0.86	8.45E-02	-0.75	1.38E-01

RUMGNA_02325	Clp protease ClpP	2.05	5.53E-03	1.68	1.12E-02	-0.38	6.47E-01
RUMGNA_02339	Hypothetical protein			-0.27	7.61E-01	-1.58	3.67E-02
RUMGNA_02387	putative argininosuccinate synthase	-0.17	9.42E-01	1.53	9.69E-02	1.7	4.65E-02
RUMGNA_02388	argininosuccinate synthase	0.02	9.95E-01	1.72	5.10E-02	1.69	3.91E-02
RUMGNA_02389	uracil permease	-0.44	4.97E-01	1.83	6.81E-08	2.28	3.66E-12
RUMGNA_02426	antirestriction protein ArdA	0.58	6.12E-01	-1.25	2.86E-02	-1.83	9.93E-04
RUMGNA_02450	TetR family transcriptional regulator	0.41	4.66E-01	-1.12	2.45E-04	-1.52	1.33E-07
RUMGNA_02458	Crp/Fnr family transcriptional regulator	0.35	5.81E-01	-1.33	2.96E-05	-1.69	5.49E-08
RUMGNA_02462	Putative nucleoside hydrolase	-0.57	2.49E-01	1.21	1.49E-04	1.78	2.01E-09
RUMGNA_02463	Inosine-uridine preferring nucleoside hydrolase	-0.49	3.56E-01	1.17	3.14E-04	1.66	3.41E-08
RUMGNA_02465	radical SAM protein	-0.79	1.27E-01	0.93	1.55E-02	1.72	3.93E-07
RUMGNA_02491	cytidylate kinase-like family protein	0.75	1.51E-01	-2.52	3.83E-15	-3.27	5.05E-25
RUMGNA_02507	helix-turn-helix domain-containing protein	0.42	4.32E-01	-1.28	1.36E-05	-1.71	2.09E-09
RUMGNA_02517	Hypothetical protein	1.15	3.90E-01	-0.94	2.58E-01	-2.09	6.49E-03
RUMGNA_02518	DUF1697 domain-containing protein	0.47	4.97E-01	-1.26	6.05E-04	-1.73	1.17E-06
RUMGNA_02536	Hypothetical protein	0.62	3.27E-01	-1.27	1.33E-03	-1.89	2.57E-07
RUMGNA_02537	L-ribulose-5-phosphate 3-epimerase	0.58	2.95E-01	-1.41	4.77E-05	-1.99	1.10E-09
RUMGNA_02538	DeoR/GlpR transcriptional regulator	0.72	1.97E-01	-1.6	1.20E-05	-2.32	1.72E-11
RUMGNA_02539	PTS lactose transporter subunit IIB	0.74	1.80E-01	-1.91	3.67E-08	-2.65	3.67E-15
RUMGNA_02548	deoxyribose-phosphate aldolase	-1.44	3.47E-02	-1.52	7.13E-03	-0.08	9.15E-01
RUMGNA_02556	phosphoribosylformylglycinamidine cyclo-ligase	0.84	4.74E-01	-0.76	3.31E-01	-1.6	1.41E-02
RUMGNA_02566	sugar phosphate isomerase/epimerase			1.65	7.04E-02	1.9	2.65E-02
RUMGNA_02567	LysR family transcriptional regulator	-0.68	3.09E-01	2.14	2.97E-07	2.82	2.22E-12
RUMGNA_02575	PTS system, beta-glucoside-specific, IIABC component	0.36	6.46E-01	-1.22	1.84E-03	-1.58	2.00E-05
RUMGNA_02593	Hypothetical protein	0.74	1.22E-01	-1.95	3.20E-10	-2.68	2.46E-19
RUMGNA_02608	Hypothetical protein	-0.02	9.92E-01	2.7	1.12E-09	2.72	5.42E-10
RUMGNA_02620	Hypothetical protein	-2.39	1.47E-04	-0.67	3.57E-01	1.72	3.77E-03
RUMGNA_02650	aminoglycoside phosphotransferase	-0.53	5.04E-01	-2.64	4.86E-11	-2.11	1.91E-07
RUMGNA_02653	50S ribosomal protein L17	-1.01	3.53E-02	0.65	1.26E-01	1.67	4.05E-06
RUMGNA_02654	DNA-directed RNA polymerase subunit alpha	-0.96	6.68E-02	0.62	1.62E-01	1.58	2.15E-05
RUMGNA_02655	30S ribosomal protein S4	-0.97	7.57E-02	0.64	1.59E-01	1.61	2.55E-05
RUMGNA_02656	30S ribosomal protein S11	-0.86	1.67E-01	0.78	9.27E-02	1.64	4.25E-05
RUMGNA_02657	30S ribosomal protein S13	-0.92	1.23E-01	0.75	1.02E-01	1.67	2.24E-05
RUMGNA_02658	50S ribosomal protein L36	-0.71	2.79E-01	0.86	5.98E-02	1.57	8.51E-05
RUMGNA_02659	translation initiation factor IF-1	-0.73	3.99E-01	1.17	3.12E-02	1.9	8.06E-05
RUMGNA_02660	RNA-binding protein	-0.89	2.56E-01	1.02	6.50E-02	1.91	7.76E-05
RUMGNA_02661	adenylate kinase	-0.56	4.05E-01	1.19	3.17E-03	1.75	2.19E-06
RUMGNA_02662	preprotein translocase subunit SecY	-0.9	1.78E-01	1	3.92E-02	1.9	7.50E-06
RUMGNA_02663	50S ribosomal protein L15	-0.91	1.81E-01	0.96	5.46E-02	1.87	1.74E-05
RUMGNA_02664	50S ribosomal protein L30	-0.83	2.97E-01	1.06	5.63E-02	1.89	9.28E-05

RUMGNA_02665	30S ribosomal protein S5	-0.99	1.78E-01	0.92	9.05E-02	1.92	4.81E-05
RUMGNA_02666	50S ribosomal protein L18	-1.05	1.67E-01	0.98	8.10E-02	2.03	3.26E-05
RUMGNA_02667	50S ribosomal protein L6	-1.1	1.21E-01	0.98	6.92E-02	2.09	8.96E-06
RUMGNA_02668	30S ribosomal protein S8	-1.06	1.35E-01	0.95	7.50E-02	2.02	1.45E-05
RUMGNA_02669	type Z 30S ribosomal protein S14	-0.96	2.09E-01	1.02	6.63E-02	1.98	4.38E-05
RUMGNA_02670	50S ribosomal protein L5	-1.09	1.27E-01	0.97	7.34E-02	2.06	1.20E-05
RUMGNA_02671	50S ribosomal protein L24	-0.97	1.80E-01	1	5.89E-02	1.98	1.94E-05
RUMGNA_02672	50S ribosomal protein L14	-1.15	9.33E-02	0.87	1.14E-01	2.02	1.73E-05
RUMGNA_02673	30S ribosomal protein S17	-1.09	1.50E-01	1.03	6.89E-02	2.12	1.63E-05
RUMGNA_02674	50S ribosomal protein L29	-0.89	3.04E-01	1.14	5.48E-02	2.03	9.28E-05
RUMGNA_02675	50S ribosomal protein L16	-0.95	1.94E-01	1.04	5.21E-02	1.99	1.94E-05
RUMGNA_02676	30S ribosomal protein S3	-1.14	8.31E-02	0.96	6.77E-02	2.1	3.96E-06
RUMGNA_02677	50S ribosomal protein L22	-1.08	1.19E-01	1.03	5.01E-02	2.11	4.00E-06
RUMGNA_02678	30S ribosomal protein S19	-1.13	1.00E-01	0.87	1.09E-01	2	1.74E-05
RUMGNA_02679	50S ribosomal protein L2	-1.07	1.15E-01	0.88	9.60E-02	1.95	1.73E-05
RUMGNA_02680	50S ribosomal protein L23	-1.06	1.36E-01	0.91	9.01E-02	1.97	2.16E-05
RUMGNA_02681	50S ribosomal protein L4	-1.03	1.36E-01	0.95	6.63E-02	1.98	1.11E-05
RUMGNA_02682	50S ribosomal protein L3	-0.94	1.82E-01	1.1	3.21E-02	2.03	6.00E-06
RUMGNA_02683	30S ribosomal protein S10	-0.97	1.53E-01	1.18	1.62E-02	2.16	8.24E-07
RUMGNA_02690	MerR family transcriptional regulator	0.87	9.86E-02	-0.74	7.08E-02	-1.61	7.28E-06
RUMGNA_02691	NanK	0.55	6.16E-01	-1.42	1.49E-02	-1.97	2.35E-04
RUMGNA_02692	NanA	1.14	1.78E-01	-1.66	4.85E-03	-2.8	1.50E-07
RUMGNA_02693	NanE	0.97	2.11E-01	-1.71	1.12E-03	-2.68	2.80E-08
RUMGNA_02694	RgNanH	0.9	2.41E-01	-1.82	2.91E-04	-2.72	4.79E-09
RUMGNA_02695	Hypothetical protein	0.89	3.14E-01	-1.93	5.17E-04	-2.82	4.50E-08
RUMGNA_02696	ABC transporter permease	1.02	2.24E-01	-1.8	1.27E-03	-2.82	3.86E-08
RUMGNA_02697	ABC transporter permease	1.02	1.45E-01	-1.53	1.79E-03	-2.54	1.14E-08
RUMGNA_02698	ABC transporter solute-binding protein	1.26	2.80E-02	-1.44	2.23E-03	-2.7	2.04E-10
RUMGNA_02704	PTS beta-glucoside transporter subunit EIIBCA	0.41	6.90E-01	-1.41	3.47E-03	-1.82	9.54E-05
RUMGNA_02726	pyrroline-5-carboxylate reductase	-0.72	2.70E-01	-1.65	7.08E-05	-0.93	2.92E-02
RUMGNA_02727	anaerobic ribonucleoside-triphosphate reductase activating protein	-1.04	1.63E-01	-1.55	2.73E-03	-0.51	3.68E-01
RUMGNA_02728	ABC transporter substrate-binding protein or Maltose-binding periplasmic proteins/domains	-9.04	3.75E-123	-8.75	2.60E-115	0.29	5.94E-01
RUMGNA_02729	ABC transporter permease	-9	9.14E-104	-8.25	7.32E-87	0.76	1.30E-01
RUMGNA_02730	sugar ABC transporter permease	-8.7	2.11E-84	-8.16	5.15E-74	0.54	3.43E-01
RUMGNA_02731	DNA-binding response regulator	-5.04	1.92E-65	-3.55	2.57E-32	1.49	2.54E-06
RUMGNA_02732	two-component sensor histidine kinase	-4.74	2.78E-70	-3.3	5.36E-34	1.44	5.62E-07
RUMGNA_02733	sugar ABC transporter substrate-binding protein	-4.24	3.55E-48	-3.02	2.44E-24	1.22	1.19E-04
RUMGNA_02734	ribonucleoside triphosphate reductase	-1.86	1.35E-09	-1.15	3.57E-04	0.7	3.05E-02

RUMGNA_02757	Hypothetical protein	0.2	9.22E-01	-3.71	8.38E-10	-3.91	1.29E-09
RUMGNA_02758	V-type ATP synthase subunit I	0.51	7.79E-01	-2.37	5.32E-03	-2.88	2.30E-04
RUMGNA_02759	V-type ATP synthase subunit K	0.99	5.54E-01	-1.61	9.90E-02	-2.6	2.56E-03
RUMGNA_02760	Hypothetical protein	0.92	5.84E-01	-1.56	1.10E-01	-2.48	4.00E-03
RUMGNA_02761	V-type ATP synthase subunit C	0.97	5.69E-01	-1.27	2.18E-01	-2.25	1.18E-02
RUMGNA_02762	V-type ATP synthase subunit F	0.89	6.16E-01	-1.4	1.71E-01	-2.29	1.10E-02
RUMGNA_02763	V-type ATP synthase subunit A	1.09	5.04E-01	-1.24	2.31E-01	-2.34	8.58E-03
RUMGNA_02765	V-type ATP synthase subunit D	0.76	6.89E-01	-1.29	2.14E-01	-2.05	2.41E-02
RUMGNA_02766	Hypothetical protein	0.66	6.12E-01	-0.98	1.85E-01	-1.64	1.35E-02
RUMGNA_02774	gfo/Idh/MocA family oxidoreductase	0.24	7.30E-01	-1.37	5.73E-06	-1.61	1.15E-07
RUMGNA_02792	transcription elongation factor GreA	-0.74	2.37E-01	0.83	6.45E-02	1.57	5.77E-05
RUMGNA_02803	glycerol dehydrogenase	-0.36	8.57E-01	1.33	1.53E-01	1.68	4.90E-02
RUMGNA_02854	Hypothetical protein	0.1	9.40E-01	-1.98	3.41E-06	-2.08	3.80E-06
RUMGNA_02859	Hypothetical protein	-0.62	3.98E-01	1.38	2.59E-03	2	2.47E-06
RUMGNA_02867	DUF177 domain-containing protein	-0.54	3.91E-01	0.97	1.34E-02	1.51	1.74E-05
RUMGNA_02877	Hypothetical protein	2.14	3.46E-05	-1.12	3.63E-02	-3.26	1.32E-12
RUMGNA_02878	V-type ATP synthase subunit C	2.08	4.57E-05	-1.01	5.98E-02	-3.09	9.46E-12
RUMGNA_02879	ATPase	1.97	1.18E-04	-0.78	1.63E-01	-2.74	1.57E-09
RUMGNA_02880	ATPase	1.83	5.75E-04	-0.85	1.21E-01	-2.68	6.29E-09
RUMGNA_02881	V-type ATP synthase subunit F	2.04	7.26E-05	-0.73	1.94E-01	-2.77	1.68E-09
RUMGNA_02882	V-type ATP synthase subunit E	2.08	2.97E-05	-0.81	1.30E-01	-2.89	8.49E-11
RUMGNA_02883	V-type ATP synthase subunit A	1.77	7.55E-04	-0.82	1.36E-01	-2.58	1.19E-08
RUMGNA_02884	V-type ATP synthase subunit B	1.61	3.48E-03	-0.6	3.04E-01	-2.22	2.10E-06
RUMGNA_02885	V-type ATP synthase subunit D	1.64	1.25E-03	-0.34	5.66E-01	-1.98	7.86E-06
RUMGNA_02897	Hypothetical protein	-0.43	6.71E-01	2.06	3.35E-05	2.49	1.32E-07
RUMGNA_02926	Cna B-type domain-containing protein	0.79	3.92E-01	-1.07	7.20E-02	-1.86	3.52E-04
RUMGNA_02928	HD domain-containing protein	1.06	1.64E-01	-0.95	9.48E-02	-2	4.65E-05
RUMGNA_02929	stannin unstructured linker	0.79	3.32E-01	-1.08	4.87E-02	-1.86	1.05E-04
RUMGNA_02930	Hypothetical protein	0.38	7.05E-01	-1.16	1.98E-02	-1.54	8.22E-04
RUMGNA_02933	Hypothetical protein	0.72	3.91E-01	-1.18	2.48E-02	-1.91	4.80E-05
RUMGNA_02935	Hypothetical protein			1.73	4.80E-02	1.63	5.31E-02
RUMGNA_02957	RNA polymerase subunit sigma-70	-0.77	1.94E-01	1.44	5.22E-04	2.21	1.06E-08
RUMGNA_02958	DUF4179 domain-containing protein	-0.51	4.76E-01	1.56	7.52E-05	2.07	2.28E-08
RUMGNA_02959	DNA polymerase III subunit epsilon	-0.28	5.63E-01	1.73	2.57E-14	2	4.73E-19
RUMGNA_02976	Hypothetical protein	-0.13	9.23E-01	1.54	1.11E-03	1.67	1.67E-04
RUMGNA_02986	Hypothetical protein	-1	2.46E-01	0.69	2.97E-01	1.69	2.03E-03
RUMGNA_02987	Hypothetical protein	-0.61	5.46E-01	2.05	1.31E-04	2.65	1.33E-07
RUMGNA_02988	Hypothetical protein	-0.55	7.11E-01	2.04	7.02E-03	2.59	2.13E-04
RUMGNA_02992	Hypothetical protein	0.03	9.88E-01	1.92	2.03E-03	1.88	1.35E-03
RUMGNA_03004	ECF transporter S component	2.96	6.15E-06	2.05	1.49E-03	-0.9	2.01E-01

RUMGNA_03005	cobalt transport protein or energy-coupling factor transporter transmembrane protein EcfT	2.73	5.56E-05	2.15	1.24E-03	-0.58	4.47E-01
RUMGNA_03006	ABC transporter ATP-binding protein	2.56	4.27E-05	2.24	2.10E-04	-0.32	6.69E-01
RUMGNA_03007	ABC transporter ATP-binding protein	2.21	8.87E-04	1.91	2.20E-03	-0.3	7.07E-01
RUMGNA_03013	ribokinase	2.56	2.16E-06	2.54	9.82E-07	-0.03	9.74E-01
RUMGNA_03014	ribonucleoside hydrolase RihC	1.64	9.86E-03	1.61	4.11E-03	-0.02	9.76E-01
RUMGNA_03016	Hypothetical protein	-0.61	4.14E-01	1.34	5.92E-03	1.94	1.75E-05
RUMGNA_03020	ABC transporter permease	-0.98	1.38E-01	1.08	4.20E-02	2.06	1.18E-05
RUMGNA_03022	MarR family transcriptional regulator	0.16	8.40E-01	2.66	5.96E-19	2.5	1.16E-16
RUMGNA_03023	ABC transporter ATP-binding protein	0.25	6.93E-01	2.53	1.32E-19	2.27	6.67E-16
RUMGNA_03024	ABC transporter ATP-binding protein	0.17	7.60E-01	2.36	2.61E-25	2.19	9.20E-22
RUMGNA_03025	ABC transporter ATP-binding protein	-0.36	5.69E-01	1.96	1.16E-09	2.33	2.14E-13
RUMGNA_03026	ABC transporter permease	-0.42	4.75E-01	1.61	3.08E-07	2.03	2.64E-11
RUMGNA_03030	Rpn family recombination-promoting nuclease/putative transposase	0.53	2.93E-01	-1.07	5.90E-04	-1.6	7.12E-08
RUMGNA_03032	Hypothetical protein	0.88	2.07E-01	-1.1	2.21E-02	-1.98	6.81E-06
RUMGNA_03033	LacI family transcriptional regulator	0.77	1.42E-01	-1.42	5.31E-05	-2.2	3.91E-11
RUMGNA_03034	LacI family DNA-binding transcriptional regulator	0.87	1.76E-02	-0.85	7.65E-03	-1.73	1.04E-09
RUMGNA_03038	Hypothetical protein	-1.84	1.97E-07	-2.43	1.92E-13	-0.58	1.27E-01
RUMGNA_03039	PucR family transcriptional regulator	-1.95	5.35E-09	-2.56	5.09E-16	-0.61	9.35E-02
RUMGNA_03040	multiple sugar-binding transporter ATP-binding protein	-3.1	5.50E-33	-4.14	1.09E-58	-1.04	2.14E-04
RUMGNA_03041	MerR family transcriptional regulator	0.77	3.26E-01	-0.76	1.45E-01	-1.53	1.13E-03
RUMGNA_03051	PRD domain-containing protein	2.54	1.35E-15	2.82	1.50E-19	0.29	4.82E-01
RUMGNA_03052	PTS glucose transporter subunit IIA	2.8	1.01E-18	2.45	1.25E-14	-0.35	3.84E-01
RUMGNA_03053	PTS glucose transporter subunit IIBC	3.38	3.02E-23	3.36	4.85E-23	-0.02	9.76E-01
RUMGNA_03056	LysR family transcriptional regulator	-0.7	2.81E-01	0.91	4.95E-02	1.61	8.43E-05
RUMGNA_03058	NAD(P)H-dependent oxidoreductase subunit E	1.68	1.18E-04	-1.3	1.39E-03	-2.99	3.65E-15
RUMGNA_03059	NADH-quinone oxidoreductase subunit NuoF	2.25	1.70E-05	-0.29	6.59E-01	-2.54	1.12E-07
RUMGNA_03060	ferredoxin	2.16	4.29E-05	-0.38	5.62E-01	-2.53	1.23E-07
RUMGNA_03080	pyridoxamine kinase	0.9	3.21E-01	1.58	7.82E-03	0.68	2.90E-01
RUMGNA_03108	Hypothetical protein	0.64	3.32E-01	-1.38	3.29E-04	-2.02	1.15E-07
RUMGNA_03109	Hypothetical protein	0.59	2.84E-01	-1.09	2.61E-03	-1.68	4.09E-07
RUMGNA_03126	QueT transporter family protein	-0.93	2.09E-02	0.86	1.48E-02	1.79	7.08E-09
RUMGNA_03137	Hypothetical protein	0.25	8.75E-01	-2.2	5.68E-04	-2.45	4.68E-05
RUMGNA_03138	Hypothetical protein	0.12	9.48E-01	-2.52	3.98E-05	-2.63	7.62E-06
RUMGNA_03139	Hypothetical protein	-0.22	8.13E-01	1.44	5.86E-04	1.66	3.55E-05
RUMGNA_03140	Hypothetical protein	0.71	1.55E-01	-1.78	1.80E-08	-2.49	5.91E-16
RUMGNA_03141	Hypothetical protein	0.43	7.66E-01	-2.17	1.76E-04	-2.6	1.98E-05
RUMGNA_03142	Activase	1.25	1.13E-03	-2.35	1.91E-14	-3.61	9.22E-31
RUMGNA_03143	2-hydroxyglutaryl-CoA dehydratase	1.28	1.26E-03	-2.48	9.89E-15	-3.76	2.27E-31

RUMGNA_03144	2-hydroxyglutaryl-CoA dehydratase	2.05	3.13E-04	-1.72	5.69E-04	-3.77	9.58E-15
RUMGNA_03145	cobyrinic acid a,c-diamide synthase	1.41	1.51E-03	-1.92	1.49E-08	-3.32	3.83E-20
RUMGNA_03146	enoate reductase	1.47	1.94E-03	-2.01	4.45E-07	-3.48	3.02E-19
RUMGNA_03148	dihydropyrimidinase	0.46	5.12E-01	-1.26	1.05E-03	-1.72	1.57E-06
RUMGNA_03154	Rpn family recombination-promoting nuclease/putative transposase	0.58	1.97E-01	-1.13	1.15E-04	-1.72	1.05E-09
RUMGNA_03182	secretion protein F			-0.34	7.82E-01	-1.93	4.06E-02
RUMGNA_03186	Hypothetical protein	0.61	5.99E-01	2.44	5.75E-05	1.82	2.79E-03
RUMGNA_03187	pro-sigmaK processing inhibitor BofA			1.97	2.31E-02	2.53	1.49E-03
RUMGNA_03243	Hypothetical protein	0.24	7.67E-01	-1.43	3.79E-05	-1.66	8.61E-07
RUMGNA_03244	DUF3784 domain-containing protein	0.26	7.08E-01	-1.57	2.72E-07	-1.82	1.95E-09
RUMGNA_03245	DUF4825 domain-containing protein	0.18	8.44E-01	-1.37	2.45E-04	-1.55	1.79E-05
RUMGNA_03291	Hypothetical protein	1.91	1.84E-05	-1.88	1.03E-05	-3.78	2.63E-22
RUMGNA_03296	elongation factor G	2.12	6.22E-10	-2.02	2.03E-09	-4.14	2.56E-38
RUMGNA_03305	copper-translocating P-type ATPase	0.46	5.88E-01	-2.32	2.80E-08	-2.78	6.56E-12
RUMGNA_03306	copper-translocating P-type ATPase	0.58	4.77E-01	-2.67	2.23E-10	-3.25	2.60E-15
RUMGNA_03307	carbon starvation protein CstA	0.53	4.89E-01	-2.46	3.52E-10	-2.98	5.02E-15
RUMGNA_03316	bifunctional precorrin-2 dehydrogenase/sirohydrochlorin ferrochelatase	-0.34	7.22E-01	1.45	2.42E-03	1.79	6.80E-05
RUMGNA_03317	precorrin-8X methylmutase	-0.46	6.90E-01	1.12	6.48E-02	1.58	3.99E-03
RUMGNA_03318	adenosylcobinamide kinase	-0.19	8.50E-01	1.46	1.30E-03	1.65	1.52E-04
RUMGNA_03350	ammonium transporter	-1.69	1.94E-03	-1.93	7.84E-05	-0.24	6.96E-01
RUMGNA_03351	P-II family nitrogen regulator	-1.06	2.33E-01	-1.83	1.57E-03	-0.77	2.14E-01
RUMGNA_03357	Hypothetical protein	0.03	9.80E-01	-1.59	3.87E-06	-1.62	1.21E-06
RUMGNA_03374	Hypothetical protein	-0.44	5.95E-01	2.67	1.88E-10	3.12	4.11E-14
RUMGNA_03403	Hypothetical protein	-1.08	2.60E-01	1.88	1.18E-02	2.96	1.31E-05
RUMGNA_03411	putative fucosidase GH29	2.79	2.66E-07	0.56	4.11E-01	-2.24	2.02E-05
RUMGNA_03413	thioether cross-link-forming SCIFF peptide maturase	-0.31	6.49E-01	1.24	2.45E-04	1.55	1.16E-06
RUMGNA_03438	adenine deaminase	1.11	4.86E-01	-0.8	4.64E-01	-1.92	3.25E-02
RUMGNA_03439	adenine deaminase	1.43	3.08E-01	-0.66	5.56E-01	-2.08	1.82E-02
RUMGNA_03442	GTPase Era	-0.12	8.99E-01	-1.5	3.96E-06	-1.39	1.39E-05
RUMGNA_03444	NAD(FAD)-dependent dehydrogenase	0.38	7.98E-01	-5.77	5.27E-37	-6.15	2.94E-29
RUMGNA_03445	pyridine nucleotide-disulfide oxidoreductase	-0.21	8.75E-01	-5.89	7.18E-45	-5.68	5.82E-36
RUMGNA_03446	FAD dependent oxidoreductase	0.06	9.79E-01	-5.79	1.44E-26	-5.85	2.67E-24
RUMGNA_03447	alpha-glycosidase or neopullulanase	-3.36	8.70E-20	-0.67	1.47E-01	2.68	6.91E-13
RUMGNA_03448	maltose ABC transporter substrate-binding protein	-3.45	1.94E-30	-0.66	8.12E-02	2.79	7.20E-20
RUMGNA_03449	maltose ABC transporter permease	-3.16	6.35E-18	-0.24	6.54E-01	2.92	2.58E-15
RUMGNA_03450	Maltose transport system permease protein malF	-3.01	1.24E-16	0.06	9.27E-01	3.06	3.08E-17
RUMGNA_03454	glycerol-3-phosphate responsive antiterminator	0.28	7.83E-01	-1.31	3.57E-03	-1.59	1.92E-04
RUMGNA_03463	Hypothetical protein	-0.73	2.79E-01	0.96	5.01E-02	1.69	1.10E-04

RUMGNA_03465	Hypothetical protein			1.35	1.20E-01	1.93	1.42E-02
RUMGNA_03466	Hypothetical protein	-0.65	2.16E-01	2.28	2.79E-12	2.93	4.61E-20
RUMGNA_03467	dipeptide ABC transporter ATP-binding protein	-0.77	1.45E-01	1.46	4.77E-05	2.23	2.23E-11
RUMGNA_03468	ABC transporter ATP-binding protein	-0.75	1.78E-01	1.52	3.64E-05	2.27	3.53E-11
RUMGNA_03469	ABC transporter permease	-0.74	1.97E-01	1.61	2.45E-05	2.35	3.96E-11
RUMGNA_03470	ABC transporter permease	-0.72	2.24E-01	1.74	4.52E-06	2.46	6.34E-12
RUMGNA_03472	M3 family oligoendopeptidase	-0.91	3.65E-02	0.69	6.50E-02	1.6	7.31E-07
RUMGNA_03473	Hypothetical protein	-1.25	1.78E-01	1.03	1.74E-01	2.29	4.01E-04
RUMGNA_03486	Abi family protein	-0.03	9.84E-01	1.48	1.33E-05	1.51	4.74E-06
RUMGNA_03499	Hypothetical protein	0.92	3.97E-02	-0.86	1.70E-02	-1.78	6.18E-08
RUMGNA_03504	helix-turn-helix domain-containing protein	0.96	4.69E-01	-0.73	3.61E-01	-1.69	2.16E-02
RUMGNA_03511	glutamine--fructose-6-phosphate transaminase (isomerizing)	-1.55	3.41E-05	-1.06	5.16E-03	0.49	2.12E-01
RUMGNA_03522	Hypothetical protein	-0.67	2.50E-01	1	1.28E-02	1.67	3.40E-06
RUMGNA_03523	Hypothetical protein	-0.57	3.05E-01	1.28	2.89E-04	1.86	1.52E-08
RUMGNA_03567	thiamine biosynthesis protein ThiC	1.24	7.57E-02	2.13	2.69E-05	0.89	1.00E-01
RUMGNA_03573	trigger factor	-0.49	3.87E-01	1.09	1.28E-03	1.58	3.75E-07
RUMGNA_03594	ribosome-associated translation inhibitor RaiA	0.63	1.97E-01	-1.75	1.50E-08	-2.38	1.09E-15
RUMGNA_03603	Hypothetical protein	0.16	9.25E-01	2.69	8.62E-05	2.53	1.89E-04
RUMGNA_03612	alpha-galactosidase	-0.24	8.69E-01	-2.02	5.41E-04	-1.78	1.92E-03
RUMGNA_03613	ABC transporter permease	0.47	6.75E-01	-2.07	1.06E-05	-2.54	3.56E-07
RUMGNA_03615	sugar ABC transporter permease	0.11	9.21E-01	-1.85	8.84E-08	-1.96	2.80E-08
RUMGNA_03616	ABC transporter, solute-binding protein	-0.61	3.92E-01	-2.39	1.65E-10	-1.79	4.26E-06
RUMGNA_03619	carbamoyl phosphate synthase small subunit	-0.11	9.10E-01	1.5	5.17E-06	1.61	4.15E-07
RUMGNA_03683	xanthine dehydrogenase FAD-binding subunit XdhB	0.77	3.22E-01	-0.8	1.41E-01	-1.58	8.40E-04
RUMGNA_03684	anthine dehydrogenase molybdenum-binding subunit XdhA	1.18	8.85E-02	-0.48	4.39E-01	-1.66	6.30E-04
RUMGNA_03685	amidohydrolase	2.07	7.95E-08	0.29	5.66E-01	-1.79	1.81E-06
RUMGNA_03738	Hypothetical protein	-0.49	7.53E-01	1.47	7.31E-02	1.96	9.56E-03
RUMGNA_03752	Transposase and inactivated derivatives	0.46	7.15E-01	-1.58	4.14E-03	-2.04	2.80E-04
RUMGNA_03753	IS66 family transposase	0.64	3.22E-01	-1.14	2.75E-03	-1.78	2.72E-06
RUMGNA_03757	putative transposase	1.02	2.35E-01	-0.65	2.62E-01	-1.67	2.33E-03
RUMGNA_03762	Hypothetical protein	0.64	2.97E-01	-1	1.14E-02	-1.64	8.89E-06
RUMGNA_03763	Hypothetical protein	0.84	1.58E-01	-0.7	9.71E-02	-1.54	6.29E-05
RUMGNA_03765	Hypothetical protein	0.75	2.27E-01	-1.71	1.15E-05	-2.45	5.50E-11
RUMGNA_03766	ABC transporter, ATP-binding protein	0.65	2.05E-01	-1.05	1.78E-03	-1.7	9.13E-08
RUMGNA_03775	Hypothetical protein	0.41	5.64E-01	-1.5	3.78E-05	-1.91	3.41E-08
RUMGNA_03777	Hypothetical protein	0.63	3.38E-01	-1.2	3.74E-03	-1.83	2.00E-06
RUMGNA_03787	N-acetylmuramoyl-L-alanine amidase	-0.94	1.60E-01	1	5.51E-02	1.94	2.60E-05
RUMGNA_03804	quinolinate synthase NadA	-0.88	1.78E-01	0.71	1.53E-01	1.59	1.86E-04

RUMGNA_03811	orotate phosphoribosyltransferase	0.31	7.05E-01	-2.67	1.23E-13	-2.99	7.66E-17
RUMGNA_03815	DNA topoisomerase 4 subunit B	-0.9	1.36E-01	0.86	5.84E-02	1.77	8.26E-06
RUMGNA_03816	DNA topoisomerase 4 subunit A	-0.9	4.90E-02	0.99	7.57E-03	1.89	9.50E-09
RUMGNA_03833	putative fucosidase GH29	-0.6	2.94E-01	0.91	1.96E-02	1.51	1.80E-05
RUMGNA_03845	Hypothetical protein	0.11	9.39E-01	2.1	5.48E-05	1.98	7.95E-05
RUMGNA_03849	guanylate kinase	-0.59	4.30E-01	1.08	1.78E-02	1.67	4.57E-05
RUMGNA_03850	multidrug efflux pump VmrA	-0.9	4.84E-01	1.65	4.65E-02	2.54	5.77E-04
RUMGNA_03851	sugar ABC transporter permease			2.06	2.08E-02	2.97	2.33E-04
RUMGNA_03852	sugar ABC transporter permease	-1.2	1.42E-01	1.93	4.15E-03	3.13	3.22E-07
RUMGNA_03853	ABC transporter substrate-binding protein	-1.4	2.75E-02	1.23	3.51E-02	2.63	2.31E-07
RUMGNA_03854	Hypothetical protein	-1.25	1.12E-01	1.09	8.44E-02	2.33	1.99E-05
RUMGNA_03856	sn-glycerol-3-phosphate ABC transporter ATP-binding protein UgpC	-0.85	5.24E-01	1	2.42E-01	1.85	1.27E-02
RUMGNA_03862	ABC transporter ATP-binding protein	0.66	3.37E-02	-0.89	3.47E-04	-1.55	9.92E-12
RUMGNA_03877	Hypothetical protein	0.24	7.99E-01	-2.52	5.39E-11	-2.76	3.92E-13
RUMGNA_03892	Hypothetical protein	1.55	5.10E-05	-0.56	1.81E-01	-2.11	7.16E-10
RUMGNA_03893	DUF2500 domain-containing protein	1.44	6.89E-05	-0.36	3.80E-01	-1.8	3.74E-08
RUMGNA_03894	helix-turn-helix domain-containing protein	1.27	2.12E-03	-0.27	5.68E-01	-1.55	1.64E-05
RUMGNA_03902	serine/arginine repetitive matrix protein 2	-0.8	4.32E-01	0.89	2.08E-01	1.68	5.93E-03
RUMGNA_03906	antitoxin HicB	-1.69	3.18E-02	0.59	4.66E-01	2.28	2.30E-04
RUMGNA_03917	IS4 family transposase	-1.07	2.04E-01	0.46	5.24E-01	1.53	8.14E-03
RUMGNA_03958	CdaR family transcriptional regulator	0.24	7.90E-01	-1.47	1.19E-04	-1.71	5.90E-06
RUMGNA_03959	GntP family permease	2.14	5.53E-08	-0.13	8.16E-01	-2.27	1.42E-09
RUMGNA_03960	glycerate kinase	1.77	1.49E-03	-0.35	5.73E-01	-2.13	1.23E-05
RUMGNA_03961	Hypothetical protein	2.41	8.40E-07	0.42	4.58E-01	-1.99	2.45E-05
RUMGNA_03962	Hypothetical protein	1.45	5.30E-04	-0.18	7.34E-01	-1.62	1.24E-05

*Condition A vs Condition B: genes with an up-regulated transcription in Condition A (Log2FC>1.5 and padj<0.05) are highlighted in red while those with an up-regulated transcription in Condition B (Log2FC<-1.5 and padj<0.05) are highlighted in green.

**Log2FC: Log2(Fold change in transcription level)

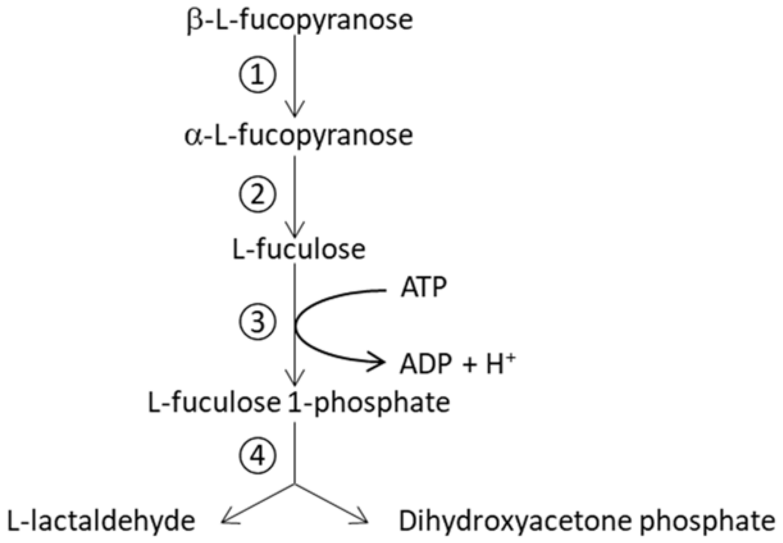

Figure S1: Schematic of fucose catabolism in bacteria
The steps are catalyzed by ①, L-fucose mutarotase (FucU); ②, L-fucose isomerase (FucI); ③, L-fuculokinase (FucK); ④, L-fuculose-1-phosphate aldolase (FucA). In *R. gnavus* ATCC 29149, these enzymes are encoded by RUMGNA_01071, RUMGNA_01070, RUMGNA_01069 and RUMGNA_01020. The genes encoding these enzymes are absent in the *R. bromii* L2-63 genome.

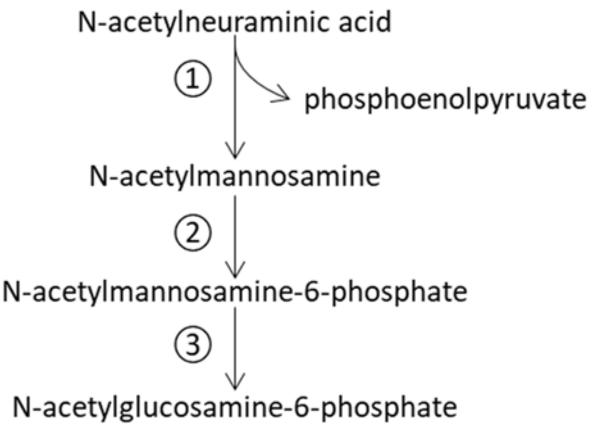

Figure S2: Schematic of sialic acid catabolism in bacteria
The steps are catalyzed by ①, N-acetylneuraminic acid lyase (NanA); ②, N-acetylmannosamine kinase (NanK); ③, N-acetylmannosamine-6-phosphate epimerase (NanE). In *R. gnavus* ATCC 29149, these enzymes are encoded by RUMGNA_02692, RUMGNA_02691 and RUMGNA_02693. The genes encoding these enzymes are absent in *R. bromii* L2-63 genome.

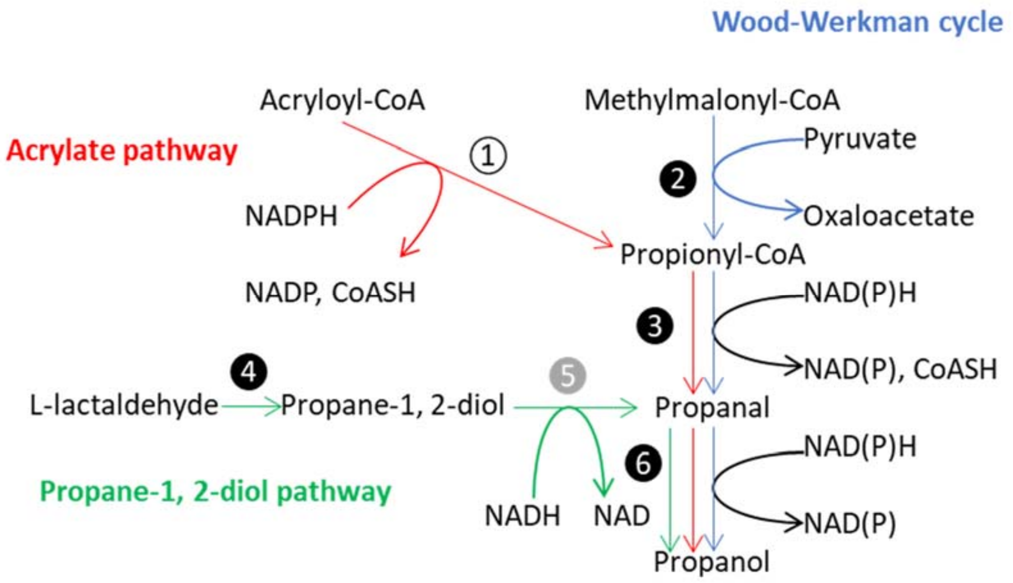

Figure S3: Propanol synthesis pathways in bacteria

Three propanol synthesis pathways have been described in bacteria. The enzymes involved in these pathways are numbered 1 to 6. White circle, enzyme absent in both *R. bromii* L2-63 and *R. gnavus* ATCC 29149; black circles, enzymes present in both *R. bromii* L2-63 and *R. gnavus* ATCC 29149; grey circle, enzyme present in *R. gnavus* ATCC 29149 only.

The first enzyme required in the acrylate pathway, in red, is the acryloyl-CoA reductase (①) which is absent in both *R. bromii* L2-63 and *R. gnavus* ATCC 29149. The Wood-Werkman cycle, in blue, requires the action of 3 successive enzymes: methylmalonyl-CoA carboxytransferase (❷) encoded by L2-63_01076 in *R. bromii* L2-63 and RUMGNA_00341 in *R. gnavus* ATCC 29149, propanal dehydrogenase (❸) encoded by L2-63_00391 in *R. bromii* L2-63 and RUMGNA_01022 in *R. gnavus* ATCC 29149 and propanol dehydrogenase (PduQ) (❻) encoded by L2-63_01124 in *R. bromii* L2-63 and RUMGNA_01033 in *R. gnavus* ATCC 29149. For the propanediol pathway in green, two enzymes are needed in addition to ❻: lactaldehyde reductase (❹) encoded by RUMGNA_02449 in *R. gnavus* ATCC 29149 and propanediol dehydratase (5) which contains 3 sub-units, PduCDE, encoded in *R. gnavus* ATCC 29149 by RUMGNA_01191, 01190 and 01189, respectively. In *R. bromii* L2-63, although L2-63_01124 is predicted to encode PduQ, the predicted protein also has high homology with ❹; however, *R. bromii* L2-63 lacks genes coding for PduCDE, ruling out the presence of a propanediol pathway in this bacterium.
